# Lichen Xanthones as Models for New Antifungal Agents

**DOI:** 10.3390/molecules23102617

**Published:** 2018-10-12

**Authors:** Diana I. S. P. Resende, Patrícia Pereira-Terra, Ângela S. Inácio, Paulo Martins da Costa, Eugénia Pinto, Emília Sousa, Madalena M. M. Pinto

**Affiliations:** 1 Laboratory of Organic and Pharmaceutical Chemistry (LQOF), Department of Chemical Sciences, Faculty of Pharmacy, University of Porto, Rua de Jorge Viterbo Ferreira, 228, 4050-313 Porto, Portugal; dresende@ff.up.pt (D.I.S.P.R.); madalena@ff.up.pt (M.M.M.P.); 2 Interdisciplinary Centre of Marine and Environmental Research (CIIMAR), Terminal de Cruzeiros do Porto de Leixões, Av. General Norton de Matos s/n, 4450-208 Matosinhos, Portugal; tichaterra@gmail.com (P.P.-T.); angelainacio@gmail.com (Â.S.I.); pmcosta@icbas.up.pt (P.M.d.C.); 3 Laboratory of Microbiology, Department of Aquatic Production, Institute of Biomedical Sciences Abel Salazar (ICBAS), University of Porto, Rua de Jorge Viterbo Ferreira, 228, 4050-313 Porto, Portugal; 4 Laboratory of Microbiology, Department of Biological Sciences, Faculty of Pharmacy, University of Porto, Rua de Jorge Viterbo Ferreira, 228, 4050-313 Porto, Portugal

**Keywords:** xanthones, synthesis, chlorination, antifungal activity, antibacterial activity

## Abstract

Due to the emergence of multidrug-resistant pathogenic microorganisms, the search for new antimicrobial compounds plays an important role in current medicinal chemistry research. Inspired by lichen antimicrobial xanthones, a series of novel chlorinated xanthones was prepared using five chlorination methods (Methods A–E) to obtain different patterns of substitution in the xanthone scaffold. All the synthesized compounds were evaluated for their antimicrobial activity. Among them, 3-chloro-4,6-dimethoxy-1-methyl-9*H*-xanthen-9-one **15** showed promising antibacterial activity against *E. faecalis* (ATCC 29212 and 29213) and *S. aureus* ATCC 29213. 2,7-Dichloro-3,4,6-trimethoxy-1-methyl-9*H*-xanthen-9-one **18** revealed a potent fungistatic and fungicidal activity against dermatophytes clinical strains (*T. rubrum*, *M. canis*, and *E. floccosum* (MIC = 4–8 µg/mL)). Moreover, when evaluated for its synergistic effect for *T. rubrum*, compound **18** exhibited synergy with fluconazole (ΣFIC = 0.289). These results disclosed new hit xanthones for both antibacterial and antifungal activity.

## 1. Introduction

**Figure 1:**
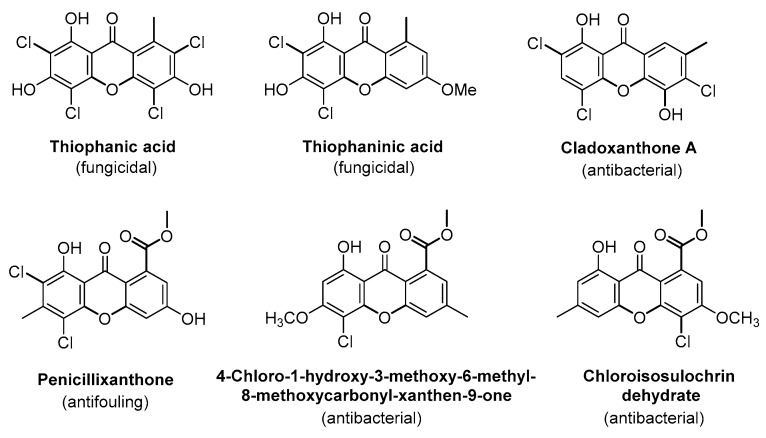
Natural chlorinated xanthones with antimicrobial activities.

Bacterial and fungal infections constitute a serious challenge due to the increasing number of multidrug resistant organisms that consequently can lead to treatment failure. The discovery of new antimicrobial drugs which can overcome problems of resistance to current anti-infective drug therapies is urgent and requires efforts in industry and scientific research communities [1]. The rapidly evolving recognition that a significant number of natural products used as anti-infective drugs/leads are actually produced by microbes [2] has led medicinal chemists to rediscover this traditional source of antimicrobial agents. Xanthones are a well-known class of secondary metabolites found in a restricted assembly of higher plants, fungi, and lichens [3]. Over the preceding decade, more than one hundred of xanthones of lichen sources were identified [4], but only a limited number have been investigated for their bioactivities [5]. Particularly, chlorinated lichen xanthones have been found attractive for their antibacterial and antifungal activities, such is the case of thiophanic and thiophaninic acids with potent fungicidal effects [6], and of cladoxanthone A with antibacterial effects towards *Staphylococcus minimoides* [7] (Figure 1). Other interesting chlorinated xanthones have been recently isolated from marine organisms. For example, penicillixanthone isolated from a marine-derived fungus *Aspergillus terreus* from the gorgonian coral *Echinogorgia aurantiaca* exhibited potent antifouling activity against larvae of the barnacle *Balanus amphitrite* [8]. Two other chlorinated metabolites, 4-chloro-1-hydroxy-3-methoxy-6-methyl-8-methoxycarbonyl-xanthen-9-one and chloroisosulochrin dehydrate were isolated from the extract of the endophytic fungus *Penicillium citrinum* HL-5126 from the mangrove *Bruguiera sexangula*; however, these revealed low antibacterial activity with MIC values of 50 μM [9].

The total synthesis of these metabolites can be quite complex, involving several steps to achieve the intricate substitution pattern. Although a number of natural chlorinated lichen xanthones, including thiophanic acid [10,11,12] have already their total synthesis described [5,13,14,15,16], these natural products can serve also as models in order to explore structure–activity relationship and improve their biological activities.

Inspired by the molecules of chlorinated natural xanthones, in the present paper we focused on the design and synthesis of new chlorinated derivatives with different patterns of substitution for their antimicrobial activity evaluation against seven Gram-positive and Gram-negative bacteria strains and five yeast and filamentous fungi strains.

## 2. Results and Discussion

### 2.1. Chemistry

The introduction of one or more chlorine atoms into the xanthone scaffold can be achieved either by junction of chlorinated building blocks of by aromatic chlorination of the xanthone core. Generally, the chlorination of aromatic compounds is achieved with molecular Cl_2_. Although the use of chlorine gas has some drawbacks regarding toxicity and hazardousness, ecofriendly procedures involving the in situ generation of Cl_2_ based on the use of NaCl/*p*-TsOH/NCS in aqueous media have already been described [17]. On the other hand, in the last years, a number of green procedures based on the generation of the electrophilic reagent Cl^+^ by an ecofriendly oxidation of chlorine ions was also reported in the literature. These include the use of benign oxidants like dimethyldioxirane (DMD), potassium peroxymono-sulfate (Oxones^®^) or NaCl, aqueous H_2_O_2_ and acetic acid [18]. Other methods use harsh conditions like neat thionyl chloride [19] or sulfuryl chloride in tetrahydrofuran [11].

**Scheme 1:**
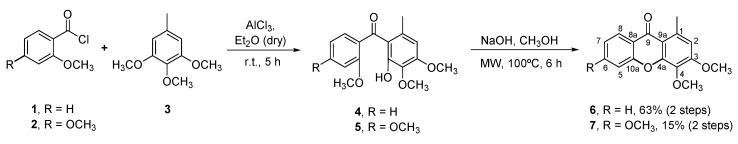
Synthesis of 3,4-dimethoxy-1-methyl-9*H*-xanthen-9-one (**6**) and 3,4,6-trimethoxy-1-methyl-9*H*-xanthen-9-one (**7**).

In order to explore the synthesis of thiophanic acid analogues, two starting materials 3,4-dimethoxy-1-methyl-9*H*-xanthen-9-one (**6**) and 3,4,6-trimethoxy-1-methyl-9*H*-xanthen-9-one (**7**) were synthesized and further submitted to chlorination. 3,4-Dimethoxy-1-methyl-9*H*-xanthen-9-one (**6**) was prepared in good yield (Scheme 1) via a previously described method [20,21]. Friedel–Crafts acylation of 1,2,3-trimethoxy-5-methylbenzene (**3**) by 2-methoxybenzoyl chloride (**1**), using aluminum chloride as acid catalyst gives the benzophenone intermediate **4**. Further nucleophilic addition followed by elimination of methanol under basic conditions and microwave irradiation produced the desired 3,4-dimethoxy-1-methyl-9*H*-xanthen-9-one (**6**).

The chlorination of 3,4-dimethoxy-1-methyl-9*H*-xanthen-9-one (**6**) was performed by using different methods in order to explore the chlorination pattern (Table 1). Method A consisted in the use of neat SOCl_2_ at room temperature for seven days [19]. It was possible to observe the formation of three different products: 1-(chloromethyl)-3,4-dimethoxy-9*H*-xanthen-9-one (**8**), 3-chloro-4-methoxy-1-methyl-9*H*-xanthen-9-one (**9**), and 3-hydroxy-4-methoxy-1-methyl-9*H*-xanthen-9-one (**10**), with 60% recovery of unreacted **6**, but none of them resulted from aromatic chlorination. Instead, probably due do the presence of water in the reaction media, demethylation and subsequent substitution occurred at C-3. Also, chlorination of the methyl group was observed possibly through a radical mechanism. Interestingly, when the temperature was raised to 40 °C (Method B) the formation of the same three products **8**, **9**, and **10** was observed with comparable yields, with 31% recovery of unreacted **6**. A larger amount of decomposition or secondary products that we were unable to isolate were also detected. The third methodology, a simple and efficient aromatic chlorination using NaCl/*p*-TsOH/NCS in aqueous media under mild conditions (Method C) [17], gave 2-chloro-3,4-dimethoxy-1-methyl-9*H*-xanthen-9-one (**11**) in 26% yield and 52% recovery of unreacted **6**. The development of the reaction proceeds through NCS reacting with NaCl to give Cl_2_, in the presence of *p*-TsOH, reacting after that with the substrate to furnish the chlorinated product.

In order to improve the reactivity of the substrate and to induce chlorination on ring B, 3,4,6-trimethoxy-1-methyl-9*H*-xanthen-9-one (**7**) was prepared according to the methodology previously described for **6** in Scheme 1. As expected, when using thionyl chloride (Methods A–C, Table 2), deprotection/substitution products are obtained. At room temperature, chlorination occurred preferentially at C-2 giving compound **12**, with traces of 2,3-dichloro-4,6-dimethoxy-1-methyl-9*H*-xanthen-9-one (**13**) and 1-(chloromethyl)-3,4,6-trimethoxy-9*H*-xanthen-9-one (**14**) were also detected with 65% of recovered starting material. When the reaction was performed at 40 °C, a deprotection at C-3 was observed and subsequent chlorination forming **15**, along with chlorination at position 2, giving **13**. As detected at room temperature, using low temperature also enabled the formation of 1-(chloromethyl)-3,4,6-trimethoxy-9*H*-xanthen-9-one (**14**), with 45% of starting material recovered. Interestingly, when the reaction mixture was refluxed, it was observed the formation of a large amount of decomposition products that we were unable to isolate or identify, with the total consumption of compound **7**. Compounds **13** and **15** resulting from deprotection/substitution were also formed, along with 3,6-dichloro-4-methoxy-1-methyl-9*H*-xanthen-9-one (**16**). The fourth method (Method D: NaCl/*p*-TsOH/NCS in aqueous media) resulted in the formation of compounds **12** and **14** with 40% recovery of unreacted **7**. In order to expand this library of compounds, a fifth method (Method E) was employed: a simple and ecofriendly procedure based on the use of sodium halides, aqueous hydrogen peroxide and acetic acid [18]. With this methodology, chlorination occurred preferentially on position C-2, forming 2-chloro-3,4,6-trimethoxy-1-methyl-9*H*-xanthen-9-one (**12**) and the corresponding 2,5- (**17**) and 2,7-dichlorinated (**18**) derivatives. Moreover, two new mono derivatives were also obtained: 5-chloro (**19**) and 7-chloro (**20**).

A series of one- and two-dimensional NMR experiments and high-resolution mass spectrometry were used to confirm the structures of all new products and to assign unequivocally the position of the substituents (Supplementary information, Figures S1–S32). As an example, ^1^H-NMR spectra of 2,7-dichloro-3,4,6-trimethoxy-1-methyl-9*H*-xanthen-9-one **18** (Figure S25) presented two aromatic singlets corresponding to the resonance of protons H-5 (δ = 6.97 ppm) and H-8 (δ = 8.25 ppm). These attributions were confirmed by HMBC correlations with C-8a, C-7, C-10a, and C-6 (H-5) and C-7, C-10a, C-6, and C-9 (H-8) (Figure 2).

### 2.2. Microbiology

In order to evaluate the antimicrobial activity of compounds **6**–**20** against Gram-positive and Gram-negative bacteria, an initial activity screening was performed by disk diffusion method for different reference strains and environmental multidrug-resistant strains. The results are presented in Table 3. Any of the tested compounds revealed antibacterial activity against Gram-negative bacteria. Regarding Gram-positive bacteria, compound **15** was effective with an inhibition halo of 10 mm for *E. faecalis* ATCC 29212 and 9.5 mm for *S. aureus* ATCC 29213. However, **15** was not effective when tested with either methicillin-resistant *S. aureus (*MRSA*)* or vancomycin-resistant enterococci (VRE)*.* Despite these encouraging results, it was not possible to determine a MIC for any compound in any of the strains in the range of the tested concentrations. This might be related to the fact that some compounds are poorly soluble in the culture media used for the determination of MIC, and the amount of available compound in the solution was probably lower than anticipated. Especially for hydrophobic compounds, such is the case of chlorinated xanthones, the diffusion through the agar media tends to be slower [22,23,24]. Regarding the screening for potential synergies with the multidrug-resistant bacterial strains and the tested compounds in combination with clinically relevant antibiotics, none of the compounds revealed a synergistic association with antibiotics (data not shown).

The results for the antifungal activity of the tested compounds against yeast and filamentous fungi are presented in Table 4. None of the compounds tested showed activity against *C. albicans* nor *A. fumigatus* strains. Nevertheless, compounds **8** and **9** revealed variable inhibitory effect on dermatophytes with MIC and MFC ranging from 128 to 256 (≥128) µg/mL, depending of the compounds and the species used for testing. Compound **18** revealed a potent inhibitory effect on the growth of different dermatophyte clinical strains (*T. rubrum* FF5, *M. canis* FF1, and *E. floccosum* FF9), with MIC of 8, 8, and 4 µg/mL, respectively. The fungistatic activity of **18** is paralleled by its fungicidal activity, with MFC of 8, 8, and 4 µg/mL, respectively.

Compounds **8**, **9**, and **18**, were also evaluated for synergistic effects for *T. rubrum*. Synergy was observed with fluconazole for compound **18** (Mean ∑FIC = 0.289).

**Figure 3:**
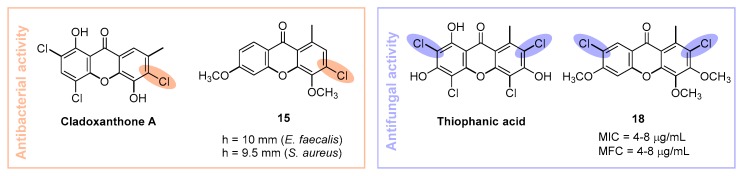
Structure-activity relationship.

Concerning structure–activity relationship (SAR) analysis, the obtained results were consistent with data previously reported for some natural products (Figure 3). For antibacterial activity, the chlorine atom at C-3 seems to have some influence since 3-chloro-4,6-dimethoxy-1-methyl-9*H*-xanthen-9-one **15** showed promising antibacterial activity against *E. faecalis* (ATCC 29212) and *S. aureus* ATCC 29213.

Regarding antifungal activity, SAR suggests that the presence of a chlorine atom at C-2, C-3, C-5, or C-7 plays an important role towards this activity. Interestingly, the fact that only 2,7-dichloro-3,4,6-trimethoxy-1-methyl-9*H*-xanthen-9-one **18**, with chlorine atoms at both C-2 and C-7, exhibited potent antifungal activity suggests that their joint presence may be required for this effect, similarly to the natural product thiophanic acid.

## 3. Materials and Methods

### 3.1. Chemistry

#### 3.1.1. General

All reagents and solvents were purchased from TCI (Tokyo Chemical Industry Co. Ltd., Chuo-ku, Tokyo, Japan), Acros Organics (Geel, Belgium), Sigma-Aldrich (Sigma-Aldrich Co. Ltd., Gillinghan, UK), or Alfa Aesar (Thermo Fisher GmbH, Kandel, Germany) and had no further purification process. Solvents were evaporated using rotary evaporator under reduced pressure, Buchi Waterchath B-480. Microwave (MW) reactions were performed using an Ethos MicroSYNTH 1600 Microwave Labstation from Milestone (Thermo Unicam, Portugal). The internal reaction temperature was controlled by a fiber optic probe sensor. All reactions were monitored by TLC carried out on precoated plates with 0.2 mm thickness using Merck silica gel 60 (GF254) with appropriate mobile phases and detection at 254 and/or 365 nm. Purification of the synthesized compounds was performed by chromatography flash column using Merck silica gel 60 (0.040–0.063 mm). Melting points (m.p.) were measured in a Köfler microscope (Wagner and Munz, Munich, Germany) and are uncorrected. ^1^H- and ^13^C-NMR spectra were taken in CDCl_3_ (Deutero GmbH, Kastellaun, Germany) at room temperature on Bruker Avance 300 instrument (300.13 or 500.13 MHz for ^1^H and 75.47 or 125.77 MHz for ^13^C, Bruker Biosciences Corporation, Billerica, MA, USA). Chemical shifts are expressed in δ (ppm) values relative to tetramethylsilane (TMS) as an internal reference. Coupling constants are reported in hertz (Hz). ^13^C-NMR assignments were made by 2D HSQC and HMBC experiments. HRMS mass spectra were measured on a Bruker FTMS APEX III mass spectrometer (Bruker Corporation, Billerica, MA, USA) recorded as ESI (Electrospray) mode in Centro de Apoio Cientifico e Tecnolóxico á Investigation (CACTI, University of Vigo, Pontevedra, Spain).

#### 3.1.2. Synthesis of the Starting Materials 3,4-Dimethoxy-1-methyl-9*H*-xanthen-9-one (**6**) and 3,4,6-Trimethoxy-1-methyl-9*H*-xanthen-9-one (**7**)

##### Synthesis of the Benzophenone Intermediates **4** and **5**

Anhydrous aluminum chloride (18.29 g, 0.137 mol) was added to a dry ether solution (200 mL) of the appropriate 2-methoxybenzoyl chloride **1** and **2** (0.066 mmol) and 3,4,5-trimethoxytoluene (**3**, 10.00 g, 0.055 mmol). The resulting two-phase, deep orange mixture was stirred at room temperature for 5 h. The reaction was monitored using *n*-hexane/ethyl acetate in a proportion of 7:3. The solvent was evaporated off under reduced pressure and the viscous residue was poured into water. The aqueous suspension was acidified with hydrochloric acid 5 M and extracted with CH_2_Cl_2_. Evaporation of the dried (Na_2_SO_4_) organic layer gave an oily residue which was used in the next reaction without further purification.

##### Synthesis of 3,4-Dimethoxy-1-methyl-9*H*-xanthen-9-one (**6**) and 3,4,6-Trimethoxy-1-methyl-9*H*-xanthen-9-one (**7**)

To a mixture of the crude containing the benzophenone intermediate **4** or **5** dissolved in MeOH (240 mL) was added a solution of NaOH (48 g, 1.2 mol) in water (160 mL). The reaction mixture was placed into Teflon reactors, provided with a fiber optic probe sensor and was submitted to 2 h (5 h for **5**) of microwave (MW) irradiation at 300 W of potency and 100 °C. After cooling, the solid was filtered and washed with water (100 mL). The crude product was recrystallized from MeOH to afford 3,4-dimethoxy-1-methyl-9*H*-xanthen-9-one (**6**) as colorless needles (9.34 g, 63% yield) or 3,4,6-trimethoxy-1-methyl-9*H*-xanthen-9-one (**7**) as a fluffy white solid (0.7 g, 15% yield).

3,4-Dimethoxy-1-methyl-9*H*-xanthen-9-one (**6**): Colorless needles (9.34 g, 63% yield); m.p. 177–178 °C. ^1^H-NMR (CDCl_3_, 300.13 MHz): δ = 8.26 (dd, ^3^*J*_8,7_ = 8.1 Hz, ^4^*J*_8,6_ = 1.7 Hz, 1H, H-8), 7.67 (ddd, ^3^*J*_6,5_ = 8.4 Hz, ^3^*J*_6,7_ = 7.1 Hz, ^4^*J*_6,8_ = 1.7 Hz, 1H, H-6), 7.51 (dd, ^3^*J*_5,6_ = 8.4 Hz, ^4^*J*_5,7_ = 1.2 Hz, 1H, H-5), 7.34 (ddd, ^3^*J*_7,8_ = 8.1 Hz, ^3^*J*_7,6_ = 7.1 Hz, ^4^*J*_7,5_ = 1.2 Hz, 1H, H-7), 6.73 (1H, s, H-2), 3.99 (3H, s, 3-OCH_3_), 3.99 (3H, s, 4-OCH_3_), 2.90 (s, 3H, 1′-CH_3_) ppm. ^13^C-NMR (CDCl_3_, 75.47 MHz): δ = 178.3 (C-9), 156.2 (C-3), 155.4 (C-10a), 152.0 (C-4a), 138.3 (C-1), 134.7 (C-4), 134.2 (C-6), 126.8 (C-8), 123.9 (C-7), 122.7 (C-8a), 117.7 (C-5), 115.1 (C-9a), 111.1 (C-2), 61.7 (4-OCH_3_), 56.3 (3-OCH_3_), 23.8 (C-1′) ppm. HRMS (ESI^+^): *m*/*z* [C_16_H_14_O_4_ + H]^+^ calcd. for [C_16_H_15_O_4_]: 271.0965; found 271.0961.

3,4,6-Trimethoxy-1-methyl-9*H*-xanthen-9-one (**7**): Fluffy white solid (0.7 g, 15% yield); m.p. 145–147 °C. ^1^H-NMR (CDCl_3_, 300.13 MHz): δ = 8.17 (d, ^3^*J*_8,7_ = 8.6 Hz, 1H, H-8), 6.93–6.88 (m, 2H, H-5, H-7), 6.71 (1H, s, H-2), 3.99 (3H, s, 3-OCH_3_), 3.99 (3H, s, 4-OCH_3_), 3.92 (3H, s, 6-OCH_3_), 2.89 (s, 3H, 1′-CH_3_) ppm. ^13^C-NMR (CDCl_3_, 75.47 MHz): δ = 177.6 (C-9), 164.7 (C-6), 157.2 (C-10a), 155.8 (C-3), 152.0 (C-4a), 138.1 (C-1), 134.7 (C-4), 128.2 (C-8), 116.6 (C-8a), 115.1 (C-9a), 113.3 (C-7), 111.1 (C- 2), 100.0 (C-5), 61.7 (4-OCH_3_), 56.3 (3-OCH_3_), 56.0 (6-OCH_3_), 23.8 (C-1′) ppm. HRMS (ESI^+^): *m*/*z* [C_17_H_16_O_5_ + H]^+^ calcd. for [C_17_H_17_O_5_]: 301.1070; found 301.1067; [C_17_H_16_O_5_ + Na]^+^ calcd. for [C_17_H_16_NaO_5_]: 323.0890; found 323.0889.

#### 3.1.3. Chlorination Studies on 3,4-Dimethoxy-1-methyl-9*H*-xanthen-9-one (**6**)

Method A. 3,4-Dimethoxy-1-methyl-9*H*-xanthen-9-one (**6**, 0.3 g, 1.110 mmol) was dissolved in thionyl chloride (15 mL). The solution was stirred at room temperature, under nitrogen atmosphere, for 7 days. After this period of time, the dark red/brown solution was slowly added into a mixture of ice/water (200 mL). The precipitate was filtered off, dissolved in CH_2_Cl_2_, and washed with water. The reunited organic fractions were dried over anhydrous Na_2_SO_4_ and the solvent was evaporated under reduced pressure. Purification of the crude by preparative TLC (SiO_2_, CH_2_Cl_2_) gave 1-(chloromethyl)-3,4-dimethoxy-9*H*-xanthen-9-one (**8**) (17.0 mg, 5% yield), 3-hydroxy-4-methoxy-1-methyl-9*H*-xanthen-9-one (**9**) (5.6 mg, 2% yield), and 3-chloro-4-methoxy-1-methyl-9*H*-xanthen-9-one (**10**) (42.2 mg, 12% yield), along with recovered 3,4-dimethoxy-1-methyl-9*H*-xanthen-9-one (**6**) (180.4 mg, 60%).

Method B. 3,4-Dimethoxy-1-methyl-9*H*-xanthen-9-one (**6**, 0.4 g, 1.479 mmol) was dissolved in thionyl chloride (1.5 mL). The solution was stirred at 40 °C, under nitrogen atmosphere, for 7 days. After this period of time, the dark red/brown solution was slowly added into a mixture of ice/water (200 mL). The precipitate was filtered off, dissolved in CH_2_Cl_2_, and washed with water. The reunited organic fractions were dried over anhydrous Na_2_SO_4_ and the solvent was evaporated under reduced pressure. Purification of the crude by preparative TLC (SiO_2_, CH_2_Cl_2_) gave 1-(chloromethyl)-3,4-dimethoxy-9*H*-xanthen-9-one (**8**) (27.2 mg, 6% yield), 3-hydroxy-4-methoxy-1-methyl-9*H*-xanthen-9-one (**9**) (14.9 mg, 4% yield), and 3-chloro-4-methoxy-1-methyl-9*H*-xanthen-9-one (**10**) (48.7 mg, 10% yield), along with recovered 3,4-dimethoxy-1-methyl-9*H*-xanthen-9-one (**6**) (123.2 mg, 31%).

1-(Chloromethyl)-3,4-dimethoxy-9*H*-xanthen-9-one (**8**): White solid; m.p. 173–175 °C. ^1^H-NMR (CDCl_3_, 300.13 MHz): δ = 8.29 (dd, ^3^*J*_8,7_ = 8.1 Hz, ^4^*J*_8,6_ = 1.7 Hz, 1H, H-8), 7.71 (ddd, ^3^*J*_6,5_ = 8.6 Hz, ^3^*J*_6,7_ = 7.1 Hz, ^4^*J*_6,8_ = 1.7 Hz, 1H, H-6), 7.54 (dd, ^3^*J*_5,6_ = 8.6 Hz, ^4^*J*_5,7_ = 1.1 Hz, 1H, H-5), 7.37 (ddd, ^3^*J*_7,8_ = 8.1 Hz, ^3^*J*_7,6_ = 7.1 Hz, ^4^*J*_7,5_ = 1.1 Hz, 1H, H-7), 7.19 (1H, s, H-2), 5.44 (s, 2H, CH_2_Cl), 4.05 (3H, s, 4-OCH_3_), 4.03 (s, 3H, 3-OCH_3_) ppm. ^13^C-NMR (CDCl_3_, 75.47 MHz): δ = 177.7 (C-9), 156.4 (C-3), 155.3 (C-10a), 152.0 (C-4a), 136.6 (C-4), 135.6 (C-1), 134.7 (C-6), 126.9 (C-8), 124.3 (C-7), 122.3 (C-8a), 117.8 (C-5), 114.0 (C-9a), 110.6 (C-2), 61.7 (4-OCH_3_), 56.5 (3-OCH_3_), 46.1 (CH_2_Cl) ppm. HRMS (ESI^+^): *m*/*z* [C_16_H_13_ClO_4_ + H]^+^ calcd. for [C_16_H_14_ClO_4_]: 305.05751; found 305.05796; [C_16_H_13_ClO_4_ + Na]^+^ calcd. for [C_16_H_13_ClNaO_4_]: 327.03946; found 327.04104.

3-Chloro-4-methoxy-1-methyl-9*H*-xanthen-9-one (**9**): White solid; m.p. 159–161 °C. ^1^H-NMR (CDCl_3_, 300.13 MHz): δ = 8.27 (dd, ^3^*J*_8,7_ = 8.1 Hz, ^4^*J*_8,6_ = 1.7 Hz, 1H, H-8), 7.72 (ddd, ^3^*J*_6,5_ = 8.6 Hz, ^3^*J*_6,7_ = 7.1 Hz, ^4^*J*_6,8_ = 1.7 Hz, 1H, H-6), 7.54 (dd, ^3^*J*_5,6_ = 8.6 Hz, ^4^*J*_5,7_ = 0.9 Hz, 1H, H-5), 7.39 (ddd, ^3^*J*_7,8_ = 8.1 Hz, ^3^*J*_7,6_ = 7.1 Hz, ^4^*J*_7,5_ = 0.9 Hz, 1H, H-7), 7.13 (1H, s, H-2), 4.04 (3H, s, 4-OCH_3_), 2.85 (s, 3H, 1′-CH_3_) ppm. ^13^C-NMR (CDCl_3_, 75.47 MHz): δ = 178.4 (C-9), 155.1 (C-10a), 151.9 (C-4a), 143.3 (C-4), 137.5 (C-1), 134.8 (C-6), 132.8 (C-3), 127.0 (C-2), 126.9 (C-8), 124.5 (C-7), 122.7 (C-8a), 120.1 (C-9a), 117.9 (C-5), 61.7 (4-OCH_3_), 23.0 (1-CH_3_) ppm. HRMS (ESI^+^): *m*/*z* [C_15_H_11_ClO_3_ + H]^+^ calcd. for [C_15_H_12_ClO_3_]: 275.0469; found 275.0463.

3-Hydroxy-4-methoxy-1-methyl-9*H*-xanthen-9-one (**10**): White solid; m.p. 176–178 °C. ^1^H-NMR (CDCl_3_, 300.13 MHz): δ = 8.28 (dd, ^3^*J*_8,7_ = 8.0 Hz, ^4^*J*_8,6_ = 1.7 Hz, 1H, H-8), 7.68 (ddd, ^3^*J*_6,5_ = 8.5 Hz, ^3^*J*_6,7_ = 7.1 Hz, ^4^*J*_6,8_ = 1.7 Hz, 1H, H-6), 7.48 (dd, ^3^*J*_5,6_ = 8.5 Hz, ^4^*J*_5,7_ = 1.1 Hz, 1H, H-5), 7.36 (ddd, ^3^*J*_7,8_ = 8.0 Hz, ^3^*J*_7,6_ = 7.1 Hz, ^4^*J*_7,5_ = 1.1 Hz, 1H, H-7), 6.77 (d, ^4^*J*_2,CH3_ = 0.9 Hz, 1H, H-2), 6.30 (s, 1H, 3-OH), 4.09 (3H, s, 4-OCH_3_), 2.85 (d, ^4^*J*_CH3,2_ = 0.9 Hz, 3H, 1′-CH_3_) ppm. ^13^C-NMR (CDCl_3_, 75.47 MHz): δ = 177.9 (C-9), 155.0 (C-10a), 152.9 (C-3), 151.4 (C-4a), 138.8 (C-1), 134.2 (C-6), 132.3 (C-4), 126.9 (C-8), 124.2 (C-7), 122.8 (C-8a), 117.4 (C-5), 114.9 (C-9a), 114.6 (C-2), 62.0 (4-OCH_3_), 23.5 (1-CH_3_) ppm. HRMS (ESI^+^): *m*/*z* [C_15_H_12_O_4_ + H]^+^ calcd. for [C_15_H_13_O_4_]: 257.0808; found 257.0810; [C_15_H_12_O_4_ + Na]^+^ calcd. for [C_15_H_12_NaO_4_]: 279.0628; found 279.0626;

Method C. Water (10 mL) was added to a finely crushed powder of 3,4-dimethoxy-1-methyl-9*H*-xanthen-9-one (**6**, 0.400 g, 1.480 mmol) taken in a 100 mL round-bottom flask equipped with a magnetic stirring bar at room temperature. To this was added NaCl (0.130 g, 2.220 mmol), *p*-TsOH (0.282 g, 1.480 mmol), and NCS (0.198 g, 1.480 mmol). The reaction completion was monitored with thin layer chromatography (TLC). After completion of the reaction, 5 mL of water was added to separate the precipitated mass; precipitates were filtered and dried in oven. The crude product was purified by preparative TLC (SiO_2_, CH_2_Cl_2_) to give 2-chloro-3,4-dimethoxy-1-methyl-9*H*-xanthen-9-one (**11**) (117.6 mg, 26%) as a white solid, along with recovered 3,4-dimethoxy-1-methyl-9*H*-xanthen-9-one (**6**) (117.6 mg, 26%).

2-Chloro-3,4-dimethoxy-1-methyl-9*H*-xanthen-9-one (**11**): White solid (117.6 mg, 26% yield); m.p. 165–167 °C. ^1^H-NMR (CDCl_3_, 300.13 MHz): δ = 8.27 (dd, ^3^*J*_8,7_ = 8.1 Hz, ^4^*J*_8,6_ = 1.7 Hz, 1H, H-8), 7.70 (ddd, ^3^*J*_6,5_ = 8.6 Hz, ^3^*J*_6,7_ = 7.1 Hz, ^4^*J*_6,8_ = 1.7 Hz, 1H, H-6), 7.51 (dd, ^3^*J*_5,6_ = 8.6 Hz, ^4^*J*_5,7_ = 1.1 Hz, 1H, H-5), 7.37 (ddd, ^3^*J*_7,8_ = 8.1 Hz, ^3^*J*_7,6_ = 7.1 Hz, ^4^*J*_7,5_ = 1.1 Hz, 1H, H-7), 4.08 (3H, s, 4-OCH_3_), 4.04 (3H, s, 3-OCH_3_), 3.00 (s, 3H, 1-CH_3_) ppm. ^13^C-NMR (CDCl_3_, 75.47 MHz): δ = 177.9 (C-9), 154.9 (C-10a), 153.5 (C-3), 151.1 (C-4a), 139.9 (C-4), 135.1 (C-1), 134.6 (C-6), 127.0 (C-8), 125.4 (C-2), 124.3 (C-7), 122.8 (C-8a), 117.6 (C-5), 62.1 (3-OCH_3_), 61.5 (4-OCH_3_), 18.3 (1-CH_3_) ppm. HRMS (ESI^+^): *m*/*z* [C_16_H_13_ClO_4_ + H]^+^ calcd. for [C_16_H_14_ClO_4_]: 305.05751; found 305.05710; [C_16_H_13_ClO_4_ + Na]^+^ calcd. for [C_16_H_13_ClNaO_4_]: 327.0394; found 327.0383.

#### 3.1.4. Chlorination Studies on 3,4,6-Trimethoxy-1-methyl-9*H*-xanthen-9-one (**7**)

Method A: 3,4,6-Trimethoxy-1-methyl-9*H*-xanthen-9-one (**7**, 0.1 g, 0.333 mmol) was dissolved in thionyl chloride (15 mL). The solution was stirred at room temperature, under nitrogen atmosphere, for 7 days. After this period of time, the orange/brown solution was slowly added into a mixture of ice/water (200 mL). The precipitate was filtered off, dissolved in CH_2_Cl_2_ and washed with water. The organic fractions were collected and dried over anhydrous Na_2_SO_4_ and the solvent was evaporated under reduced pressure. Purification of the crude by preparative TLC (SiO_2_, CH_2_Cl_2_) gave 2-chloro-3,4,6-trimethoxy-1-methyl-9*H*-xanthen-9-one (**12**) (7.4 mg, 7% yield), 2,3-dichloro-4,6-dimethoxy-1-methyl-9*H*-xanthen-9-one (**13**) (traces), and 1-(chloromethyl)-3,4,6-trimethoxy-9*H*-xanthen-9-one (**14**) (traces), along with recovered 3,4,6-trimethoxy-1-methyl-9*H*-xanthen-9-one (**7**) (64.5 mg, 65%).

2-Chloro-3,4,6-trimethoxy-1-methyl-9*H*-xanthen-9-one (**12**): White solid (7.4 mg, 7% yield); m.p. 165–167 °C. ^1^H-NMR (CDCl_3_, 300.13 MHz): δ = 8.17 (d, ^3^*J*_8,7_ = 8.4 Hz, 1H, H-8), 6.95-6.89 (m, 2H, H-5, H-7), 4.06 (s, 3H, 3-OCH_3_), 4.07 (3H, s, 4-OCH_3_), 3.93 (3H, s, 6-OCH_3_), 2.99 (s, 3H, 1-CH_3_) ppm. ^13^C-NMR (CDCl_3_, 75.47 MHz): δ = 177.1 (C-9), 164.9 (C-6), 156.7 (C-10a), 153.2 (C-3), 151.0 (C-4a), 139.9 (C-4), 135.0 (C-1), 128.5 (C-8), 125.3 (C-2), 117.6 (C-9a), 116.6 (C-8a), 113.7 (C-7), 99.8 (C-5), 62.1 (4-OCH_3_), 61.5 (3-OCH_3_), 56.0 (6-OCH_3_), 18.2 (1-CH_3_) ppm. HRMS (ESI^+^): *m*/*z* [C_17_H_15_ClO_5_ + Na]^+^ calcd. for [C_20_H_9_ClNaO_2_]: 339.01833; found 339.01814.

Method B: 3,4,6-Trimethoxy-1-methyl-9*H*-xanthen-9-one (**7**, 0.1 g, 0.333 mmol) was dissolved in thionyl chloride (15 mL). The solution was stirred at 40 °C, under nitrogen, for 7 days. After this period of time, the dark orange/brown solution was slowly added into a mixture of ice/water (200 mL). The precipitate was filtered off, dissolved in CH_2_Cl_2_ and washed with water. The organic fractions were collected and dried over anhydrous Na_2_SO_4_ and the solvent was evaporated under reduced pressure. Purification of the crude by preparative TLC (SiO_2_, CH_2_Cl_2_) gave 2,3-dichloro-4,6-dimethoxy-1-methyl-9*H*-xanthen-9-one (**13**) (13.4 mg, 12% yield), 1-(chloromethyl)-3,4,6-trimethoxy-9*H*-xanthen-9-one (**14**) (9.4 mg, 8% yield), and 3-chloro-4,6-dimethoxy-1-methyl-9*H*-xanthen-9-one (**15**) (5.0 mg, 5% yield), along with recovered 3,4,6-trimethoxy-1-methyl-9*H*-xanthen-9-one (**7**) (45.1 mg, 45%).

2,3-Dichloro-4,6-dimethoxy-1-methyl-9*H*-xanthen-9-one (**13**): White solid (13.4 mg, 12% yield); m.p. 210–212 °C. ^1^H-NMR (CDCl_3_, 300.13 MHz): δ = 8.16 (d, ^3^*J*_8,7_ = 8.9 Hz, 1H, H-8), 6.95 (dd, ^3^*J*_7,8_ = 8.9 Hz, ^4^*J*_7,5_ = 2.4 Hz, 1H, H-7), 6.90 (d, ^4^*J*_5,7_ = 2.4 Hz, 1H, H-5), 4.05 (s, 3H, 4-OCH_3_), 3.95 (s, 3H, 6-OCH_3_), 3.03 (s, 1H, 1-CH_3_) ppm. ^13^C-NMR (CDCl_3_, 75.47 MHz): δ = 177.0 (C-9), 165.3 (C-6), 156.6 (C-10a), 150.3 (C-4a), 144.1 (C-4), 135.5 (C-1), 132.8 (C-3), 129.3 (C-2), 128.6 (C-8), 120.3 (C-9a), 116.6 (C-8a), 114.1 (C-7), 99.8 (C-5), 61.7 (4-OCH_3_), 56.1 (6-OCH_3_), 19.0 (1-CH_3_) ppm. HRMS (ESI^+^): *m*/*z* [C_17_H_15_ClO_5_ + Na]^+^ calcd. for [C_20_H_9_ClNaO_2_]: 339.0183; found 339.0181.

1-(Chloromethyl)-3,4,6-trimethoxy-9*H*-xanthen-9-one (**14**): White solid (9.4 mg, 8% yield); m.p. 178–180 °C. ^1^H-NMR (CDCl_3_, 300.13 MHz): δ = 8.19 (d, ^3^*J*_8,7_ = 9.3 Hz, 1H, H-8), 7.17 (s, 1H, H-2), 6.95-6.90 (m, 2H, H-5, H-7), 5.45 (s, 2H, H-1′a, H-1′b), 4.04 (s, 3H, 3-OCH_3_), 4.02 (s, 3H, 4-OCH_3_), 3.93 (s, 3H, 6-OCH_3_) ppm. ^13^C-NMR (CDCl_3_, 75.47 MHz): δ = 177.6 (C-9), 165.1 (C-6), 157.0 (C-10a), 152.0 (C-4a), 143.2 (C-4), 137.5 (C-1), 132.3 (C-3), 128.4 (C-8), 127.0 (C-2), 120.2 (C-9a), 116.6 (C-8a), 113.9 (C-7), 100.0 (C-5), 61.7 (4-OCH_3_), 56.1 (6-OCH_3_), 23.0 (1-CH_3_) ppm. HRMS (ESI^+^): *m*/*z* [C_17_H_15_ClO_5_ + H]^+^ calcd. for [C_17_H_16_ClO_5_]: 335.0681; found 335.0671.

3-Chloro-4,6-dimethoxy-1-methyl-9*H*-xanthen-9-one (**15**): White solid (5.0 mg, 5% yield); m.p. 163–165 °C. ^1^H-NMR (CDCl_3_, 300.13 MHz): δ = 8.17 (dd, ^3^*J*_8,7_ = 8.3 Hz, ^4^*J*_8,5_ = 0.9 Hz, 1H, H-8), 7.11 (d, ^3^*J*_2,1-CH3_ = 0.9 Hz, 1H, H-2), 6.96-6.90 (m, 2H, H-5, H-7), 4.04 (s, 3H, 4-OCH_3_), 3.94 (s, 3H, 6-OCH_3_), 2.94 (d, ^3^*J*_1-CH3,2_ = 0.9 Hz 3H, 1-CH_3_) ppm. ^13^C-NMR (CDCl_3_, 75.47 MHz): δ = 177.6 (C-9), 165.1 (C-6), 157.0 (C-10a), 152.0 (C-4a), 143.2 (C-4), 137.5 (C-1), 132.3 (C-3), 128.4 (C-8), 127.0 (C-2), 120.2 (C-9a), 116.6 (C-8a), 113.9 (C-7), 100.0 (C-5), 61.7 (4-OCH_3_), 56.1 (6-OCH_3_), 23.0 (1-CH_3_) ppm. HRMS (ESI^+^): *m*/*z* [C_16_H_13_ClO_4_ + H]^+^ calcd. for [C_16_H_14_ClO_4_]: 305.0575; found 305.0570.

Method C: 3,4,6-Trimethoxy-1-methyl-9*H*-xanthen-9-one (**7**, 0.1 g, 0.333 mmol) was dissolved in thionyl chloride (15 mL). The solution was refluxed, under nitrogen, for 7 days. After this period of time, the dark brown solution was slowly added into a mixture of ice/water (200 mL). The precipitate was filtered off, dissolved in CH_2_Cl_2_ and washed with water. The organic fractions were collected and dried over anhydrous Na_2_SO_4_ and the solvent was evaporated under reduced pressure. Purification of the crude by preparative TLC (SiO_2_, CH_2_Cl_2_) gave 2,3-dichloro-4,6-dimethoxy-1-methyl-9*H*-xanthen-9-one (**13**) (9.1 mg, 8% yield), 3-chloro-4,6-dimethoxy-1-methyl-9*H*-xanthen-9-one (**15**) (9.9 mg, 10% yield), and 3,6-dichloro-4-methoxy-1-methyl-9*H*-xanthen-9-one (**16**) (6.0 mg, 6% yield).

3,6-Dichloro-4-methoxy-1-methyl-9*H*-xanthen-9-one (**16**): White solid (6.0 mg, 6% yield); m.p. 181–183 °C. ^1^H-NMR (CDCl_3_, 300.13 MHz): δ = 8.20 (d, ^3^*J*_8,7_ = 8.5 Hz, 1H, H-8), 7.57 (d, ^4^*J*_5,7_ = 1.9 Hz, 1H, H-5), 7.35 (dd, ^3^*J*_7,8_ = 8.5 Hz, ^4^*J*_7,5_ = 1.9 Hz, 1H, H-7), 7.15 (d, ^4^*J*_2,1-CH3_ = 0.8 Hz, 1H, H-2), 4.03 (s, 3H, 4-OCH_3_), 2.83 (d, ^4^*J*_1-CH3,2_ = 0.8 Hz, 3H, 1-CH_3_) ppm. ^13^C-NMR (CDCl_3_, 75.47 MHz): δ = 177.5 (C-9), 155.3 (C-10a), 150.3 (C-4a), 143.4 (C-4), 140.9 (C-6), 137.6 (C-1), 133.3 (C-3), 128.3 (C-8), 127.5 (C-2), 125.4 (C-7), 121.3 (C-8a), 120.1 (C-9a), 117.9 (C-5), 61.7 (4-OCH_3_), 22.9 (1-CH_3_) ppm. HRMS (ESI^+^): *m*/*z* [C_15_H_10_Cl_2_O_3_ + H]^+^ calcd. for [C_15_H_11_Cl_2_O_3_]: 309.0080; found 309.0072.

Method D: To a mixture of 3,4,6-trimethoxy-1-methyl-9*H*-xanthen-9-one (**7**, 0.1 g, 0.333 mmol) and water was added NaCl (0.029 g, 0.499 mmol), *p*-TsOH (0.063, 0.333 mmol), and NCS (0.044 g, 0.333 mmol) and the solution was stirred at room temperature for 7 days. After that period of time, 5 mL of water was added and the mixture was extracted with CH_2_Cl_2_. The organic fractions were collected and dried over anhydrous Na_2_SO_4_ and the solvent was evaporated under reduced pressure. Purification of the crude by preparative TLC (SiO_2_, CH_2_Cl_2_) gave 2-chloro-3,4,6-trimethoxy-1-methyl-9*H*-xanthen-9-one (**12**) (8.2 mg, 7% yield), and 1-(chloromethyl)-3,4,6-trimethoxy-9*H*-xanthen-9-one (**14**) (14.2 mg, 13% yield), along with recovered 3,4,6-trimethoxy-1-methyl-9*H*-xanthen-9-one (**7**) (39.6 mg, 40%).

Method E: A 30% aqueous solution of hydrogen peroxide (4.0 mL) was added to a solution of 3,4,6-trimethoxy-1-methyl-9*H*-xanthen-9-one (**7**, 1.0 g, 3.330 mmol) and NaCl (1.107 g, 19.979 mmol) in acetic acid (4.0 mL); then, the mixture was stirred at room temperature or 40 °C for 24 h, with additions of hydrogen peroxide (4.0 mL) and acetic acid (4.0 mL) at each 24 h, for a total of 7 days. At the end, the crude was treated with sodium thiosulfate and extracted with ethyl acetate (3 × 10 mL). The reunited organic fractions were collected and dried over anhydrous Na_2_SO_4_ and the solvent was evaporated under reduced pressure. Purification of the crude by preparative TLC (SiO_2_, CH_2_Cl_2_) gave 2-chloro-3,4,6-trimethoxy-1-methyl-9*H*-xanthen-9-one (**12**) (160.4 mg, 14% yield), 2,5-dichloro-3,4,6-trimethoxy-1-methyl-9*H*-xanthen-9-one (**17**) (traces), 2,7-dichloro-3,4,6-trimethoxy-1-methyl-9*H*-xanthen-9-one (**18**) (traces), 5-chloro-3,4,6-trimethoxy-1-methyl-9*H*-xanthen-9-one (**19**) (57.3 mg, 5% yield), 7-chloro-3,4,6-trimethoxy-1-methyl-9*H*-xanthen-9-one (**20**) (traces), along with recovered 3,4,6-trimethoxy-1-methyl-9*H*-xanthen-9-one (**7**) (418.8 mg, 42%).

2,5-Dichloro-3,4,6-trimethoxy-1-methyl-9*H*-xanthen-9-one (**17**): White solid (traces); m.p. 193–195 °C. ^1^H-NMR (CDCl_3_, 300.13 MHz): δ = 8.19 (d, ^3^*J*_8,7_ = 9.0 Hz, 1H, H-8), 7.03 (d, ^3^*J*_7,8_ = 9.0 Hz, 1H, H-7), 4.13 (s, 3H, 4-OCH_3_), 4.10 (s, 3H, 4-OCH_3_), 4.06 (3H, s, 6-OCH_3_), 2.99 (s, 3H, 1-CH_3_) ppm. ^13^C-NMR (CDCl_3_, 75.47 MHz): δ = 176.9 (C-9), 160.0 (C-6), 153.6 (C-3), 152.0 (C-10a), 151.1 (C-4a), 139.9 (C-4), 134.9 (C-1), 126.4 (C-8), 125.7 (C-2), 117.6 (C-5), 116.8 (C-9a), 109.4 (C-8a), 108.4 (C-7), 61.5 (3-OCH_3_, 4-OCH_3_), 57.0 (6-OCH_3_), 18.2 (1-CH_3_) ppm. HRMS (ESI^+^): *m*/*z* [C_17_H_14_Cl_2_O_5_ + H]^+^ calcd. for [C_17_H_15_Cl_2_O_5_]: 369.0291; found 369.0290.

2,7-Dichloro-3,4,6-trimethoxy-1-methyl-9*H*-xanthen-9-one (**18**): White solid (traces); m.p. 187–189 °C. ^1^H-NMR (CDCl_3_, 300.13 MHz): δ = 8.25 (s, 1H, H-8), 6.97 (s, 1H, H-5), 4.07 (s, 3H, 3-OCH_3_), 4.04 (6H, s, 4-OCH_3_, 6-OCH_3_), 2.99 (s, 3H, 1-CH_3_) ppm. ^13^C-NMR (CDCl_3_, 75.47 MHz): δ = 176.0 (C-9), 159.8 (C-6), 154.9 (C-10a), 153.3 (C-3), 151.8 (C-4a), 139.8 (C-4), 135.0 (C-1), 127.9 (C-8), 125.6 (C-9a), 120.2 (C-7), 117.2 (C-2), 116.6 (C-8a), 99.7 (C-5), 62.0 (4-OCH_3_), 61.3 (3-OCH_3_), 56.9 (6-OCH_3_), 18.1 (1-CH_3_) ppm. HRMS (ESI^+^): *m*/*z* [C_17_H_14_Cl_2_O_5_ + H]^+^ calcd. for [C_17_H_15_Cl_2_O_5_]: 369.0291; found 369.0287.

5-Chloro-3,4,6-trimethoxy-1-methyl-9*H*-xanthen-9-one (**19**): White solid (57.3 mg, 5% yield); m.p. 207–209 °C. ^1^H-NMR (CDCl_3_, 300.13 MHz): δ = 8.17 (d, ^3^*J*_8,7_ = 9.0 Hz, 1H, H-8), 6.99 (d, ^3^*J*_7,8_ = 9.0 Hz, 1H, H-7), 6.73 (s, 1H, H-2), 4.08 (s, 3H, 4-OCH_3_), 4.05 (3H, s, 6-OCH_3_), 3.99 (3H, s, 3-OCH_3_), 2.88 (s, 3H, 1-CH_3_) ppm. ^13^C-NMR (CDCl_3_, 75.47 MHz): δ = 177.3 (C-9), 159.8 (C-6), 156.3 (C-3), 152.4 (C-10a), 151.9 (C-4a), 138.0 (C-1), 135.1 (C-4), 126.1 (C-8), 117.6 (C-5), 114.4 (C-9a), 111.5 (C-2), 109.5 (C-8a), 107.9 (C-7), 61.6 (4-OCH_3_), 56.9 (6-OCH_3_), 56.4 (3-OCH_3_), 23.7 (1-CH_3_) ppm. HRMS (ESI^+^): *m*/*z* [C_17_H_15_ClO_5_ + H]^+^ calcd. for [C_17_H_16_ClO_5_]: 335.0681; found 335.0678.

7-Chloro-3,4,6-trimethoxy-1-methyl-9*H*-xanthen-9-one (**20**): White solid (traces); m.p. 234–236 °C. ^1^H-NMR (CDCl_3_, 300.13 MHz): δ = 8.22 (s, 1H, H-8), 6.98 (s, 1H, H-5), 6.72 (s, 1H, H-2), 4.02 (s, 3H, 6-OCH_3_), 3.99 (3H, s, 3-OCH_3_), 3.98 (3H, s, 4-OCH_3_), 2.87 (s, 3H, 1-CH_3_) ppm. ^13^C-NMR (CDCl_3_, 75.47 MHz): δ = 176.4 (C-9), 159.5 (C-6), 155.9 (C-3), 155.4 (C-10a), 151.7 (C-4a), 138.1 (C-1), 134.5 (C-4), 127.6 (C-8), 119.6 (C-7), 116.5 (C-8a), 114.6 (C-9a), 111.2 (C-2), 100.0 (C-5), 61.6 (4-OCH_3_), 56.8 (6-OCH_3_), 56.2 (3-OCH_3_), 23.6 (1-CH_3_) ppm. HRMS (ESI^+^): *m*/*z* [C_17_H_15_ClO_5_ + H]^+^ calcd. for [C_17_H_16_ClO_5_]: 335.0681; found 335.0672.

### 3.2. Microbiology

#### 3.2.1. Microorganism Strains and Growth Conditions

In the present study, two Gram-positive reference strains—*Staphylococcus aureus* ATCC 29213 and *Enterococcus faecalis* ATCC 29212—and two Gram-negative reference strains—*Escherichia coli* ATCC 25922 and *Pseudomonas aeruginosa* ATCC 27853—were used. Multidrug-resistant bacterial strains isolated from public buses (MRSA *S. aureus* 66/1) [25], river water (VRE *E. faecalis* B3/101) [26], and a clinical isolate (ESBL *E. coli* SA/2) were also used when an inhibition halo was detected and it was possible to determine a minimum inhibitory concentration (MIC) value for ATCC strains. Frozen stocks of all strains were grown in Mueller-Hinton agar (MH, BioKar diagnostics, Allone, France) at 37 °C. All bacterial strains were subcultured in MH agar and incubated overnight at 37 °C before each assay. For the antifungal activity screening, a yeast reference strain *Candida albicans* ATCC 10231, a filamentous fungi reference strain *Aspergillus fumigatus* ATCC 46645, and tree dermatophyte clinical strains *Trichophyton rubrum* FF5, *Microsporum canis* FF1, and *Epidermophyton floccosum* FF9 were used. Frozen stocks of all fungal strains were subcultured in Sabouraud dextrose agar (SDA, BioMèrieux, Marcy L’Etoile, France) before each test, to ensure optimal growth conditions and purity.

#### 3.2.2. Antimicrobial Susceptibility Testing

##### Antibacterial Activity

An initial screening of the antibacterial activity of the compounds was performed by the disk diffusion method as previously described [27,28]. Briefly, sterile 6 mm blank paper disks (Oxoid, Basingstoke, UK) impregnated with 15 µg of each compound were placed on MH agar plates inoculated with the bacteria. A blank disk with DMSO was used as a negative control. MH inoculated plates were incubated for 18–20 h at 37 °C. At the end of incubation, the inhibition halos where measured. The minimum inhibitory concentration (MIC) was determined for each compound, in accordance with the recommendations of the Clinical and Laboratory Standards Institute (CLSI) [29]. For each compound, a stock solution of 10 mg/mL was prepared in dimethylsulfoxide (DMSO, Alfa Aesar, Kandel, Germany). For compounds **6**, **11**, **13**, **15**–**17**, **19**, and **20**, which were less soluble in DMSO than the other compounds, a stock solution of 2 mg/mL was prepared. In the case of compounds **10**, **12**, and **18** the stock solution prepared was 1 mg/mL. Two-fold serial dilutions of the compounds were prepared in Mueller-Hinton broth 2 (MHB2, Sigma-Aldrich, St. Louis, MO, USA) within the concentration range of 0.062 to 64 µg/mL. The highest concentration tested was chosen in order to maintain DMSO in-test concentration below 1% (*v*/*v*), as recommended by the CLSI [29]. At this concentration DMSO did not affected bacterial growth. Cefotaxime (CTX) ranging between 0.031 and 16 µg/mL was used as a quality control for *E. coli* reference strain ATCC 25922. Sterility and growth controls were included in each assay. Purity check and colony counts of the inoculum suspensions were also evaluated in order to ensure that the final inoculum density closely approximates the intended number (5 × 10^5^ CFU/mL). The MIC was determined as the lowest concentration at which no visible growth was observed. The minimum bactericidal concentration (MBC) was assessed by spreading 10 µL of culture collected from wells showing no visible growth on MH agar plates. The MBC was determined as the lowest concentration at which no colonies grew after 16–18 h incubation at 37 °C. These assays were performed in duplicate.

##### Antifungal Activity

The antifungal activity of all tested compounds was evaluated against *C. albicans*, *A. fumigatus*, and *T. rubrum*. For compounds showing some activity in the dermatophyte *T. rubrum* the activity was enlarged to other genus of dermatophytes (*M. canis* and *E. floccosum*). The MIC of each compound was determined by the broth microdilution method according to CLSI guidelines (reference documents M27-A3 for yeasts [30] and M38-A2 for filamentous fungi [31]). Briefly, cell or spore suspensions were prepared in RPMI-1640 broth medium supplemented with MOPS (Sigma-Aldrich, St. Louis, MO, USA) from fresh cultures of the different strains of fungi. In the case of filamentous fungi, the inoculum was adjusted to 0.4–5 × 10^4^ CFU/mL for *A. fumigatus* ATCC 46645 and to 1–3 × 10^3^ CFU/mL for the dermatophytes. The inoculum of *C. albicans* was adjusted to 0.5–2.5 × 10^3^ CFU/mL. Two-fold serial dilutions of the compounds were prepared in RPMI-1640 broth medium supplemented with MOPS within the concentration range of 1 to 128 µg/mL, with maximum DMSO concentration not exceeding 2.5% (*v*/*v*). Sterility and growth controls were also included in each assay. The plates were incubated for 48 h at 35 °C (*C. albicans* and *A. fumigatus*) or for 5 days at 25 °C (*T. rubrum*, *M. canis* and *E. floccosum*). MICs were recorded as the lowest concentrations resulting in 100% growth inhibition in comparison to the compound-free controls. Voriconazole MIC for reference strain *Candida krusei* ATCC 6258 was used as quality control [30,31]. The results obtained were within the recommended limits. The minimum fungicidal concentration (MFC) was determined by spreading 20 µL of culture collected from wells showing no visible growth on SDA plates. The MFC was determined as the lowest concentration showing 100% growth inhibition after 48 h at 35 °C (for *C. albicans* and *A. fumigatus*) or 5 days incubation at 25 °C (*T. rubrum*, *M. canis* and *E. floccosum*). All the experiments were repeated independently at least two times.

#### 3.2.3. Antibiotic Synergy Testing

##### Antibiotic Synergy

In order to evaluate the combined effect of the compounds and clinically relevant antimicrobial drugs, a screening was conducted using the disk diffusion method, as previously described [27,28]. A set of antibiotic disks (Oxoid, Basingstoke, UK), to which the isolates were resistant, was selected: cefotaxime (CTX, 30 µg) for *E. coli* SA/2, oxacillin (OX, 1 µg) for *S. aureus* 66/1, and vancomycin (VA, 30 µg) for *E. faecalis* B3/101. Antibiotic disks alone (controls) and antibiotic disks impregnated with 15 µg of each compound were placed on MH agar plates seeded with the respective bacteria. Sterile 6 mm blank paper impregnated with 15 µg of each compound alone were also tested. A blank disk with DMSO was used as a negative control. MH inoculated plates were incubated for 18 to 20 h at 37 °C. Potential synergism was recorded when the halo of an antibiotic disk impregnated with a compound was greater than the halo of the antibiotic or compound-impregnated blank disk alone.

##### Antifungal Synergy

In order to evaluate the combined effect of the compounds and clinically relevant antifungal drugs, checkerboard assay was conducted, as previously described [32]. Fluconazole was used in a range between 0.062 and 4 µg/mL and compounds were tested in a range between their MIC and progressive two-fold dilutions. Potential synergism was recorded when the inhibitions of the combined compounds with antifungals is greater than the compounds or the antifungals alone.

Fractional inhibitory concentration (FIC) was calculated as follows: FIC of compound = MIC of compound in combination with antifungal/MIC of compound alone and FIC of antifungal = MIC of antifungal in combination with compound/MIC of antifungal alone. FIC index (∑FIC) = FIC of compound + FIC of antifungal.

∑FIC ≤ 0.5 synergy, 0.5 < ∑FIC ≤ 4 no interaction, 4 < ∑FIC antagonism.

## 4. Conclusions

A series of xanthones (**6**–**20**) was synthesized and evaluated for its antibacterial and antifungal activity. Some of the methodologies used, such as NaCl, *p*-TsOH, and NCS, were more selective and produced higher yields while others, like the use of thionyl chloride gave a higher diversity of compounds but with lower yields. The presence of the chlorine isotopic pattern in the HRMS spectra was crucial to identify the presence of one or two chlorine atoms and bidimensional NMR to disclose the position of those atoms and the substitution pattern. Although some of the compounds exhibited great potential as antibacterial or antifungal agents, the low solubility displayed by some derivatives limited further screenings. Nevertheless, compounds **15** and **18** can be used in the future as models in order to improve drug-like properties.

## Figures and Tables

**Figure 2:**
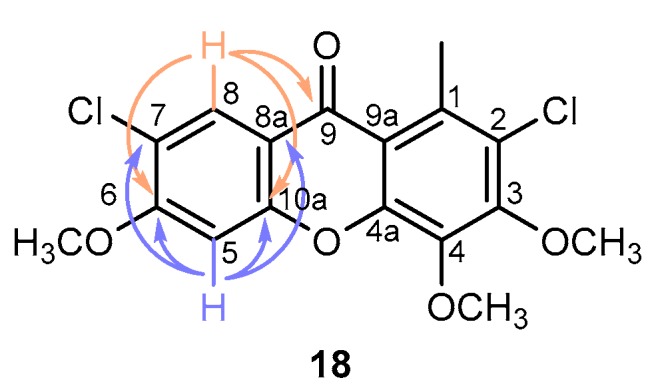
Relevant correlations of 2,7-dichloro-3,4,6-trimethoxy-1-methyl-9*H*-xanthen-9-one **18** HMBC spectra.

**Table 1:**
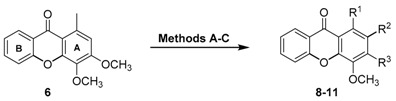
Synthesis of chlorinated derivatives of 3,4-dimethoxy-1-methyl-9*H*-xanthen-9-one (**6**).

Comp.	R^1^	R^2^	R^3^	Method	Yield (%)
**8**	CH_2_Cl	H	OCH_3_	A B	5 6
**9**	CH_3_	H	OH	A B	2 4
**10**	CH_3_	H	Cl	A B	12 12
**11**	CH_3_	Cl	OCH_3_	C	26

Method A: SOCl_2_, r.t., seven days; Method B: SOCl_2_, 40 °C, seven days; Method C: NaCl, *p*-TsOH, NCS, H_2_O, r.t., seven days.

**Table 2:**
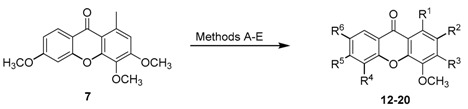
Synthesis of chlorinated derivatives of 3,4,6-trimethoxy-1-methyl-9*H*-xanthen-9-one (**7**).

Comp.	R^1^	R^2^	R^3^	R^4^	R^5^	R^6^	Method	Yield (%)
**12**	CH_3_	Cl	OCH_3_	H	OCH_3_	H	A D E	7 12 14
**13**	CH_3_	Cl	Cl	H	OCH_3_	H	A B C	Traces 12 8
**14**	CH_2_Cl	H	OCH_3_	H	OCH_3_	H	A B D	Traces 8 13
**15**	CH_3_	H	Cl	H	OCH_3_	H	B C	5 10
**16**	CH_3_	H	Cl	H	Cl	H	**C**	**6**
**17**	CH_3_	Cl	OCH_3_	Cl	OCH_3_	H	E	traces
**18**	CH_3_	Cl	OCH_3_	H	OCH_3_	Cl	E	traces
**19**	CH_3_	H	OCH_3_	Cl	OCH_3_	H	E	5
**20**	CH_3_	H	OCH_3_	H	OCH_3_	Cl	E	1

Method A: SOCl_2_, r.t, seven days; Method B: SOCl_2_, 40 °C, seven days; Method C: SOCl_2_, ∆, seven days; Method D: NaCl, *p*-TsOH, NCS, H_2_O, r.t., seven days; Method E: H_2_O_2_, AcOH, NaCl, 40 °C, seven days.

**Table 3:** Antibacterial activity of the compounds **6**–**20**. MIC and MBC ^1^ are expressed in µg/mL. Inhibition halos ^2^ are expressed in mm.

Comp.	*E. coli* ATCC 25922			*E. coli* SA/2			*P. aeruginosa* ATCC 27853			*E. faecalis* ATCC 29212			*E. faecalis* B3/101 (VRE)			*S. aureus* ATCC 29213			*S. aureus* 66/1 (MRSA)		
	Halo	MIC	MBC	Halo	MIC	MBC	Halo	MIC	MBC	Halo	MIC	MBC	Halo	MIC	MBC	Halo	MIC	MBC	Halo	MIC	MBC
**6**	0	>16	ND	0	ND	ND	0	>16	ND	0	>16	ND	0	ND	ND	0	>16	ND	0	ND	ND
**7**	0	>64	ND	0	ND	ND	0	>64	ND	0	>64	ND	0	ND	ND	0	>64	ND	0	ND	ND
**8**	0	>64	ND	0	ND	ND	0	>64	ND	0	>64	ND	0	ND	ND	0	>64	ND	0	ND	ND
**9**	0	>64	ND	0	ND	ND	0	>64	ND	0	>64	ND	0	ND	ND	0	>64	ND	0	ND	ND
**10**	0	>32	ND	0	ND	ND	0	>32	ND	0	>32	ND	0	ND	ND	0	>32	ND	0	ND	ND
**11**	0	>32	ND	0	ND	ND	0	>32	ND	0	>32	ND	0	ND	ND	0	>32	ND	0	ND	ND
**12**	0	>32	ND	0	ND	ND	0	>32	ND	0	>32	ND	0	ND	ND	0	>32	ND	0	ND	ND
**13**	0	>32	ND	0	ND	ND	0	>32	ND	0	>32	ND	0	ND	ND	0	>32	ND	0	ND	ND
**14**	0	>64	ND	0	ND	ND	0	>64	ND	0	>64	ND	0	ND	ND	0	>64	ND	0	ND	ND
**15**	0	>16	ND	0	ND	ND	0	>16	ND	10	>16	ND	0	ND	ND	**9.5**	>16	ND	0	ND	ND
**16**	0	>32	ND	0	ND	ND	0	>32	ND	0	>32	ND	0	ND	ND	0	>32	ND	0	ND	ND
**17**	0	>32	ND	0	ND	ND	0	>32	ND	0	>32	ND	0	ND	ND	0	>32	ND	0	ND	ND
**18**	0	>32	ND	0	ND	ND	0	>32	ND	0	>32	ND	0	ND	ND	0	>32	ND	0	ND	ND
**19**	0	>32	ND	0	ND	ND	0	>32	ND	0	>32	ND	0	ND	ND	0	>32	ND	0	ND	ND
**20**	0	>32	ND	0	ND	ND	0	>32	ND	0	>32	ND	0	ND	ND	0	>32	ND	0	ND	ND

^1^ MIC—minimum inhibitory concentration; MBC—minimum bactericidal concentration; ^2^ halo of partial inhibition; ND—Not determined.

**Table 4:** Antifungal activity of compounds **6**–**20**. MIC and MFC are expressed in µg/mL ^1^.

Comp.	*C. albicans* ATCC 10231		*A. fumigatus* ATCC 46645		*T. rubrum* FF5		*M. canis* FF1		*E. floccosum* FF9	
	MIC	MFC	MIC	MFC	MIC	MFC	MIC	MFC	MIC	MFC
**6**	>32	>32	>32	>32	>32	>32	ND	ND	ND	ND
**7**	>128	>128	>128	>128	>128	>128	ND	ND	ND	ND
**8**	>128	>128	>128	>128	≥128	>128	>128	>128	128	128
**9**	>128	>128	>128	>128	≥128	>128	≥128	>128	≥128	>128
**10**	>32	>32	>32	>32	>32	>32	ND	ND	ND	ND
**11**	>32	> 32	>32	>32	>32	>32	ND	ND	ND	ND
**12**	>32	>32	>32	>32	>32	>32	ND	ND	ND	ND
**13**	>128	>128	>128	>128	>128	>128	ND	ND	ND	ND
**14**	>128	>128	>128	>128	>128	>128	ND	ND	ND	ND
**15**	>32	>32	>32	>32	>32	>32	ND	ND	ND	ND
**16**	>32	>32	>32	>32	>32	>32	ND	ND	ND	ND
**17**	>32	>32	>32	>32	>32	>32	ND	ND	ND	ND
**18** ^2^	>128	>128	>128	>128	**8**	**8**	**8**	**8**	4	4
**19**	>32	>32	>32	>32	>32	>32	ND	ND	ND	ND
**20**	>32	>32	>32	>32	>32	>32	ND	ND	ND	ND

^1^ MIC—minimum inhibitory concentration; MFC—minimum fungicidal concentration; ^2^ Synergy with fluconazole.

## Supplementary Materials

The following are available on line, Figures S1–S32: NMR spectra of compounds **6**–**20**.
