# Supplementary material for: Lichen Xanthones as Models for New Antifungal Agents
Source: Molecules. 2018 Oct 12;23(10):2617. doi: 10.3390/molecules23102617 (PMC6222623; doi:10.3390/molecules23102617)
Supplement: Supplementary file 1 [file molecules-23-02617-s001.pdf]

SUPPLEMENTARY INFORMATION

Lichen xanthones as models for new antifungal agents

Diana I. S. P. Resende [1,2,†], Patrícia Pereira-Terra [2,3,†], Ângela Inácio, [2,3] Paulo Martins da Costa [2,3], Eugénia Pinto [2,4*], Emília Sousa [1,2*] and Madalena M. M. Pinto[1,2]

[1] Laboratory of Organic and Pharmaceutical Chemistry (LQOF), Department of Chemical Sciences, Faculty of Pharmacy, University of Porto, Rua de Jorge Viterbo Ferreira, 228, 4050-313 Porto, Portugal; dresende@ff.up.pt (D. I. S. P. R); esousa@ff.up.pt (E.S.); madalena@ff.up.pt (M.M.M.P.);
[2] Interdisciplinary Centre of Marine and Environmental Research (CIIMAR), Terminal de Cruzeiros do Porto
de Leixões, Av. General Norton de Matos s/n, 4450-208 Matosinhos, Portugal;
[3] Laboratory of Microbiology, Department of Aquatic Production, Institute of Biomedical Sciences Abel Salazar (ICBAS), University of Porto, Rua de Jorge Viterbo Ferreira, 228, 4050-313 Porto, Portugal; tichaterra@gmail.com (P.T); pmcosta@icbas.up.pt (P.C.); angelainacio@gmail.com (A.I.);
[4] Laboratory of Microbiology, Department of Biological Sciences, Faculty of Pharmacy, University of Porto, Rua de Jorge Viterbo Ferreira, 228, 4050-313 Porto Portugal; epinto@ff.up.pt (E.P.);
* Correspondence: epinto@ff.up.pt (E.P.), Tel.: +351220428585; esousa@ff.up.pt (E.S.), Tel.: +351220428689.
† These authors contributed equally to this work.

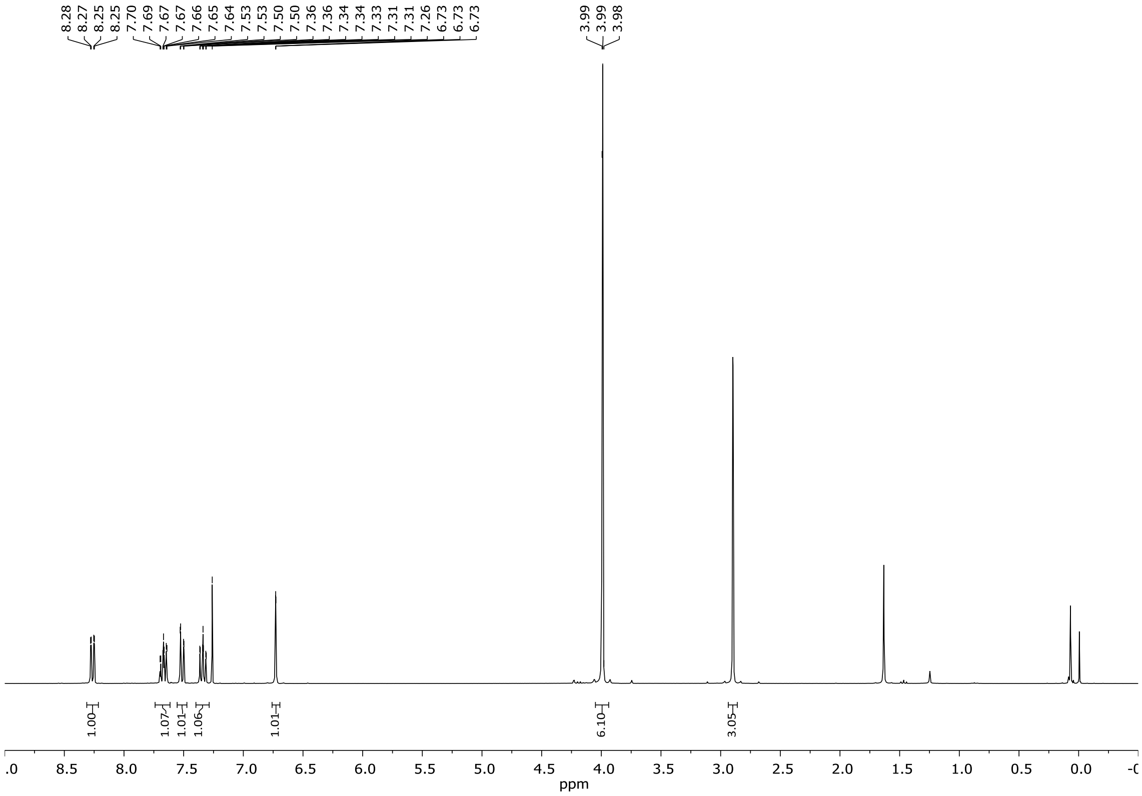

Figure S1. ^{1}H NMR spectrum of 3,4-dimethoxy-1-methyl-9*H*-xanthen-9-one (**6**) ($CDCl_3$, 300 MHz).

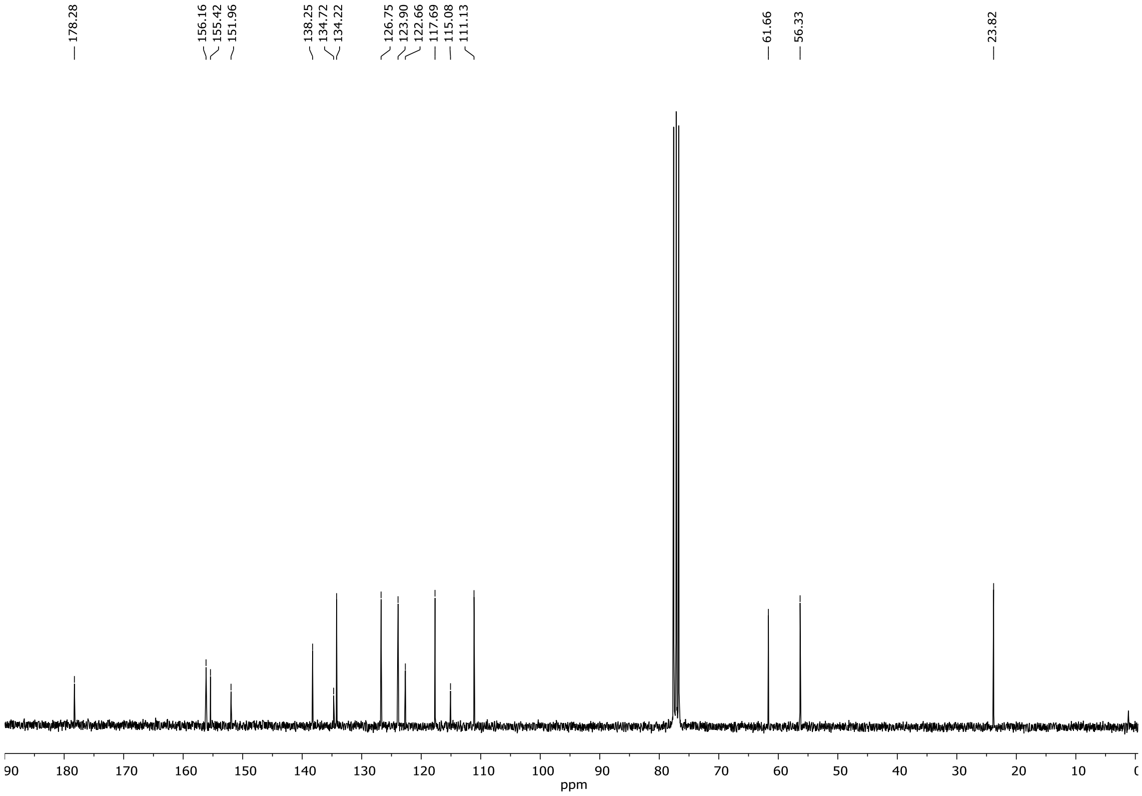

Figure S2. ^{13}C NMR spectrum of 3,4-dimethoxy-1-methyl-9*H*-xanthen-9-one (**6**) ($CDCl_3$, 300 MHz).

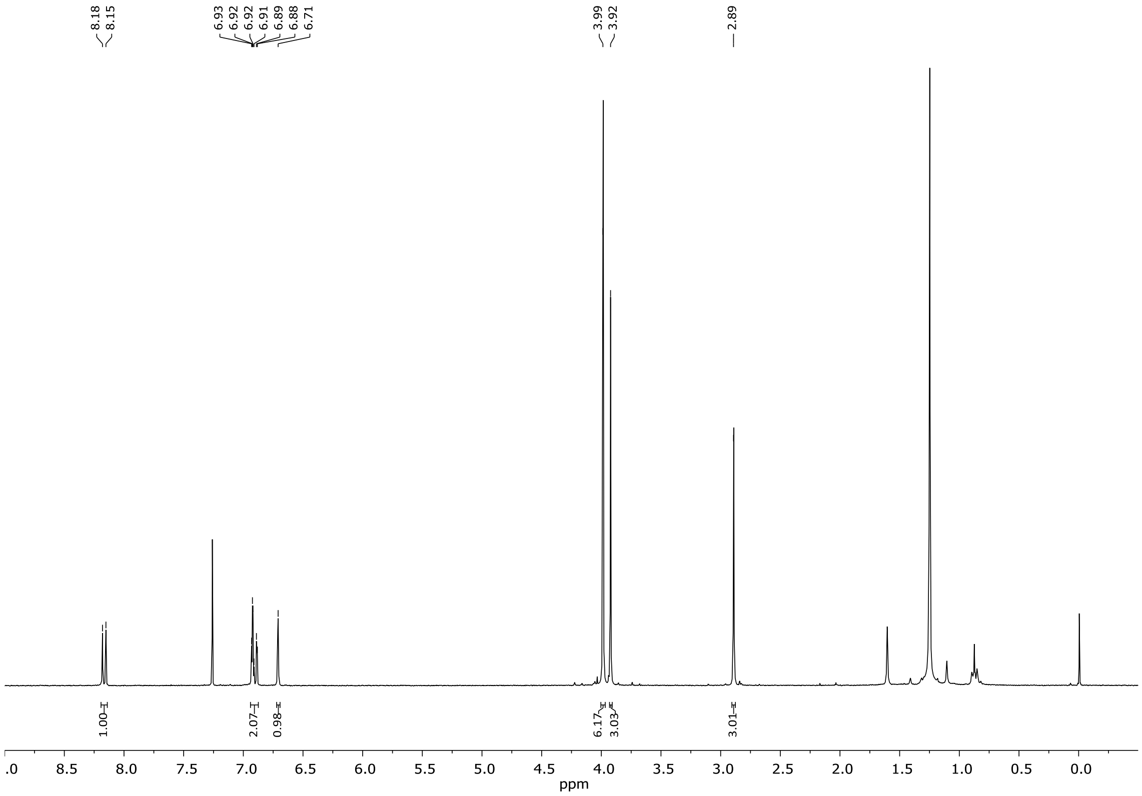

Figure S3. ^{1}H NMR spectrum of 3,4,6-trimethoxy-1-methyl-9*H*-xanthen-9-one (**7**) ($CDCl_3$, 300 MHz).

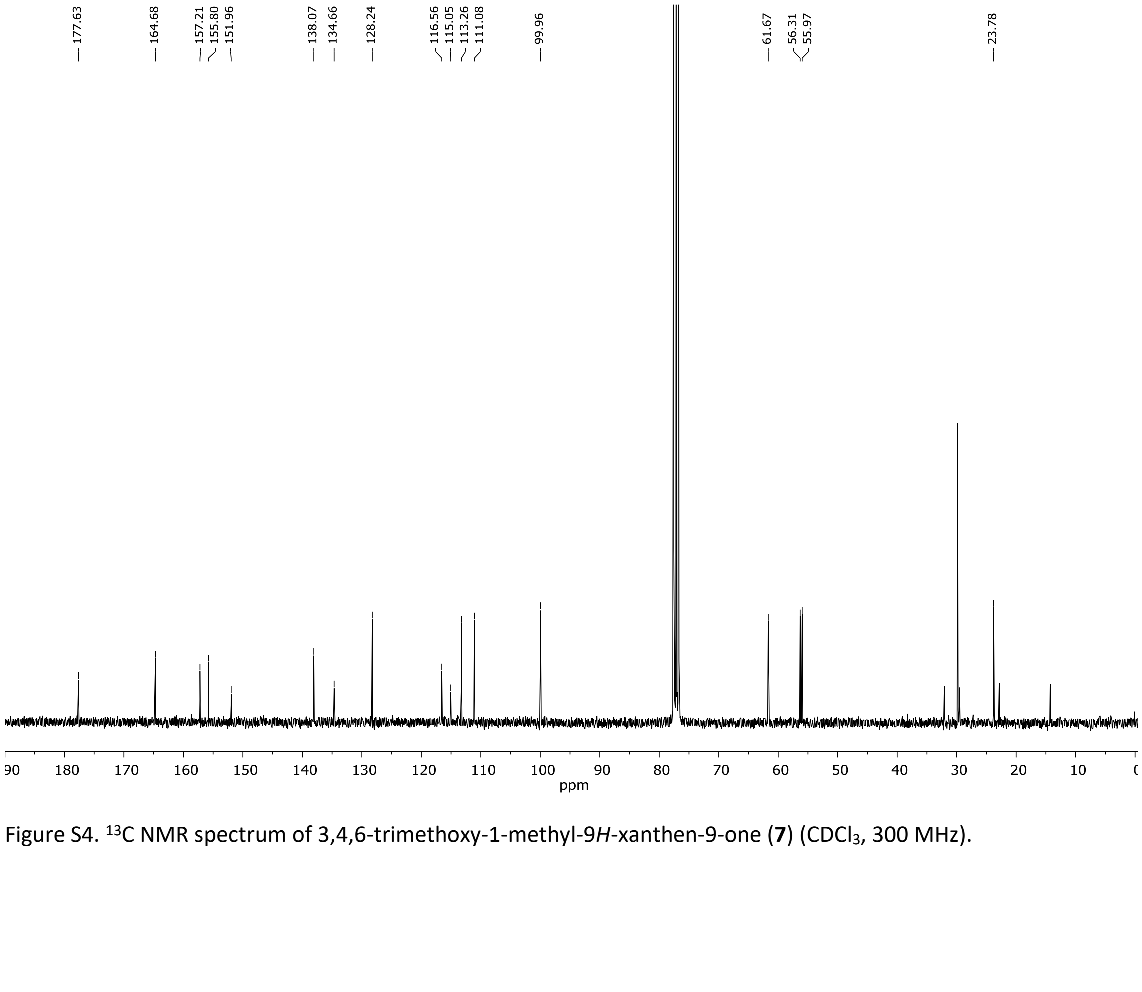

Figure S4. ^{13}C NMR spectrum of 3,4,6-trimethoxy-1-methyl-9*H*-xanthen-9-one (**7**) ($CDCl_3$, 300 MHz).

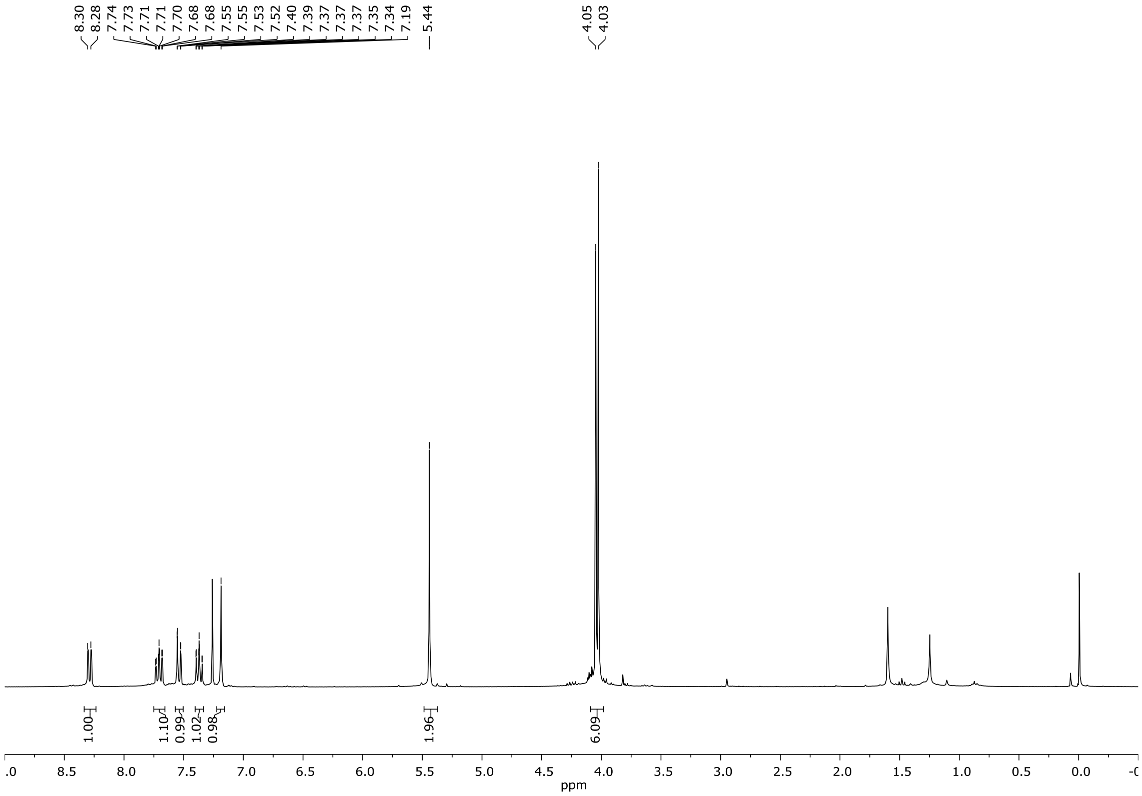

Figure S5. ^{1}H NMR spectrum of 1-(chloromethyl)-3,4-dimethoxy-9*H*-xanthen-9-one (**8**) ($CDCl_3$, 300 MHz).

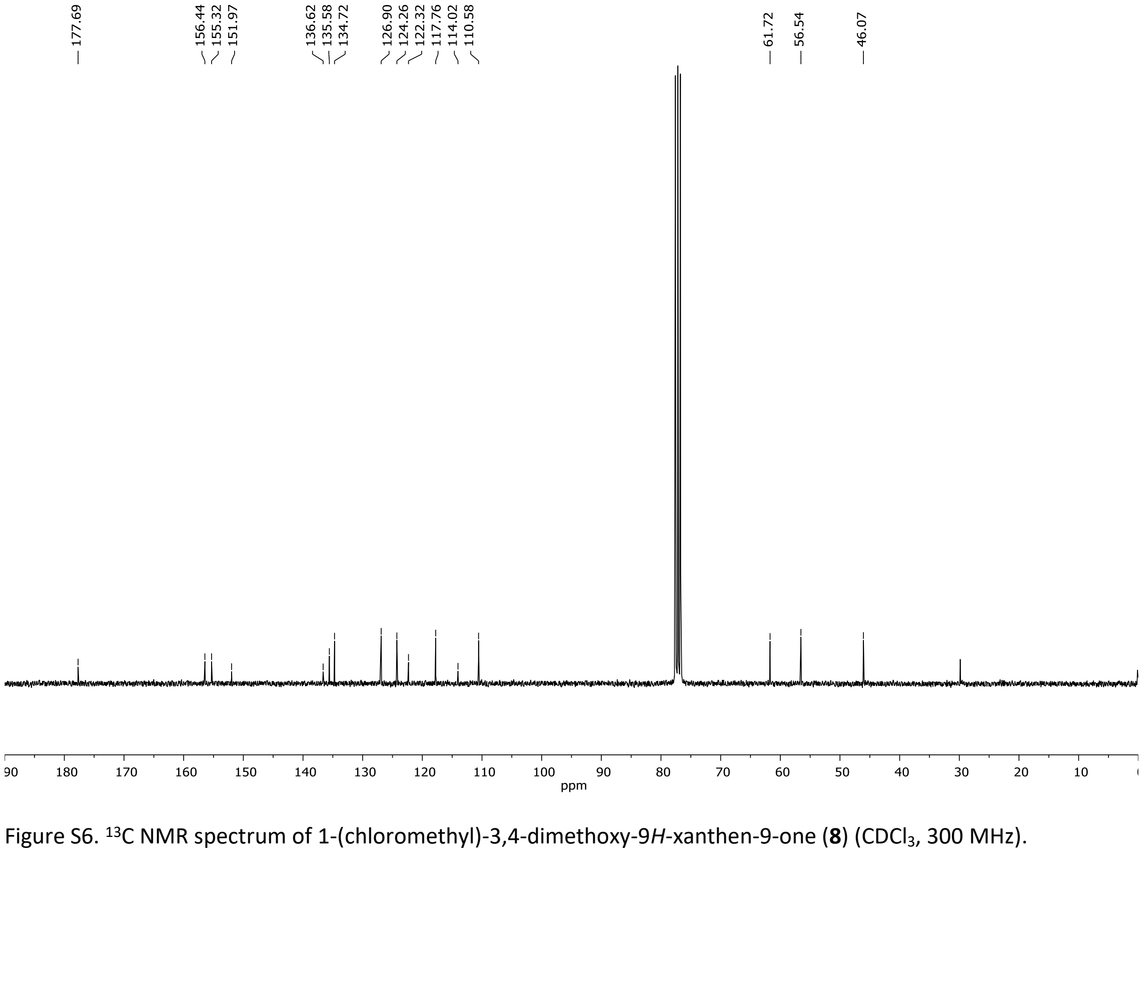

Figure S6. ^{13}C NMR spectrum of 1-(chloromethyl)-3,4-dimethoxy-9*H*-xanthen-9-one (**8**) ($CDCl_3$, 300 MHz).

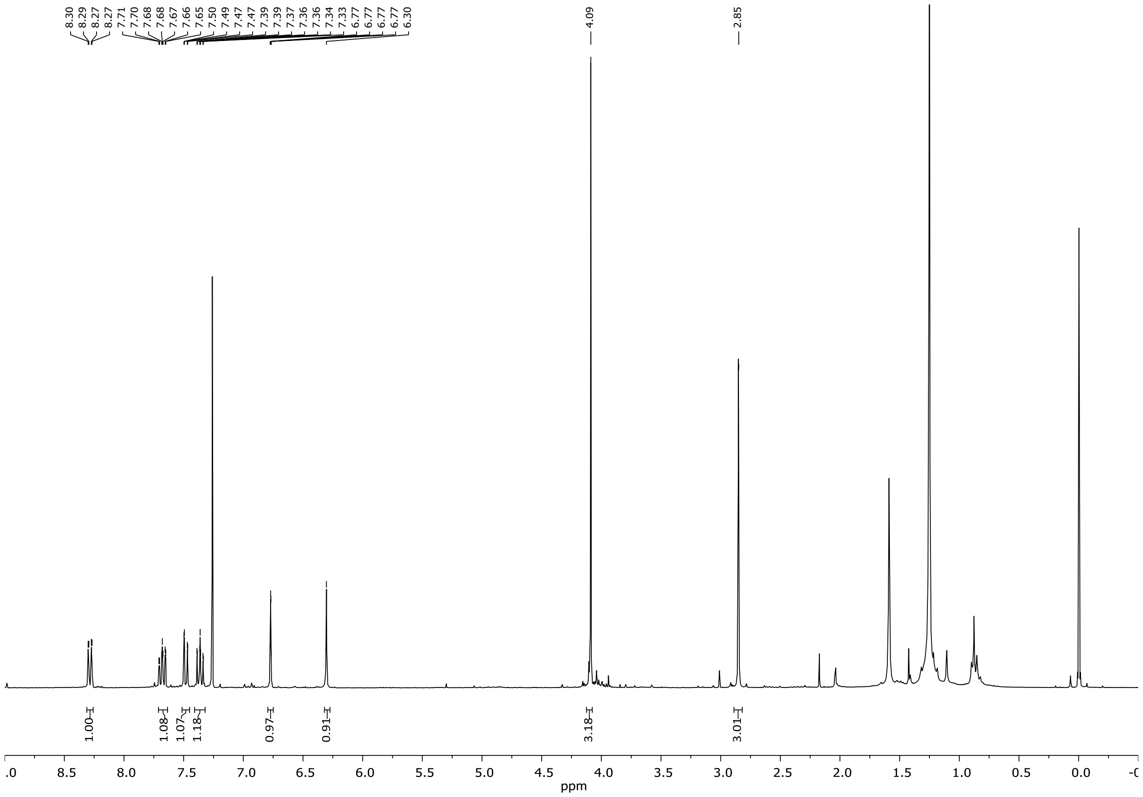

Figure S7. ^{1}H NMR spectrum of 3-chloro-4-methoxy-1-methyl-9*H*-xanthen-9-one (**9**) ($CDCl_3$, 300 MHz).

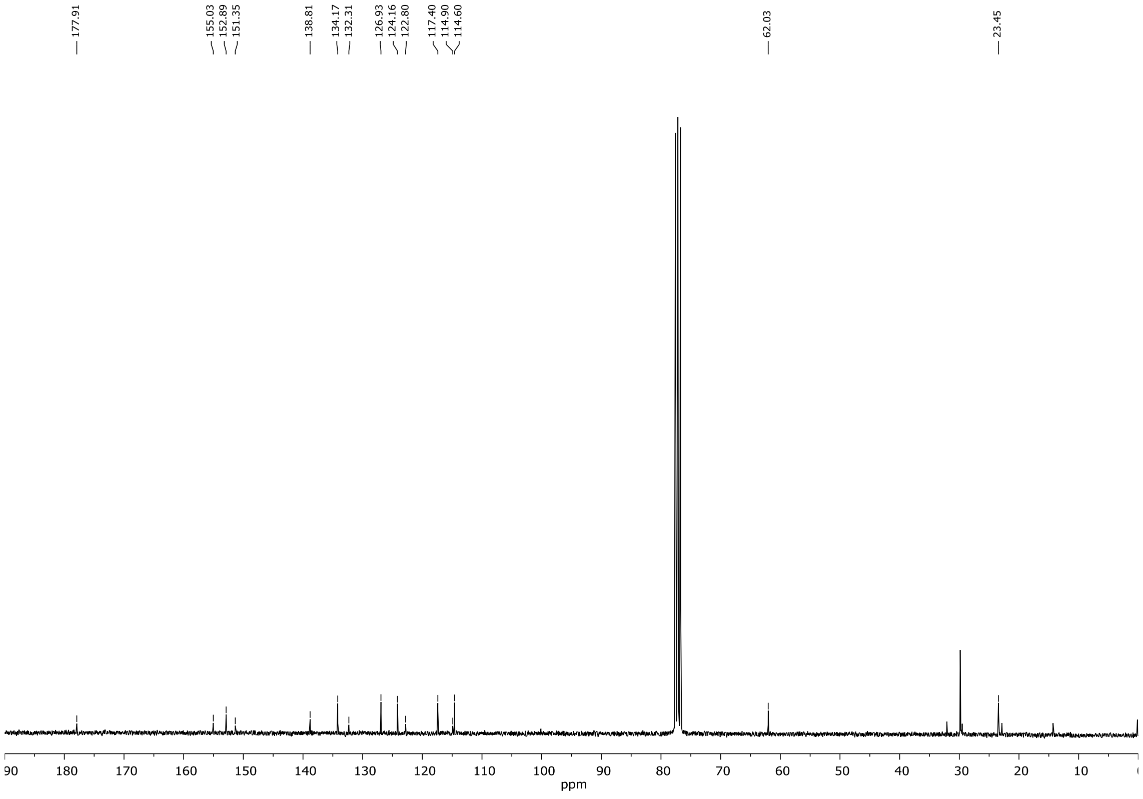

Figure S8. ^{13}C NMR spectrum of 3-chloro-4-methoxy-1-methyl-9*H*-xanthen-9-one (**9**) ($CDCl_3$, 300 MHz).

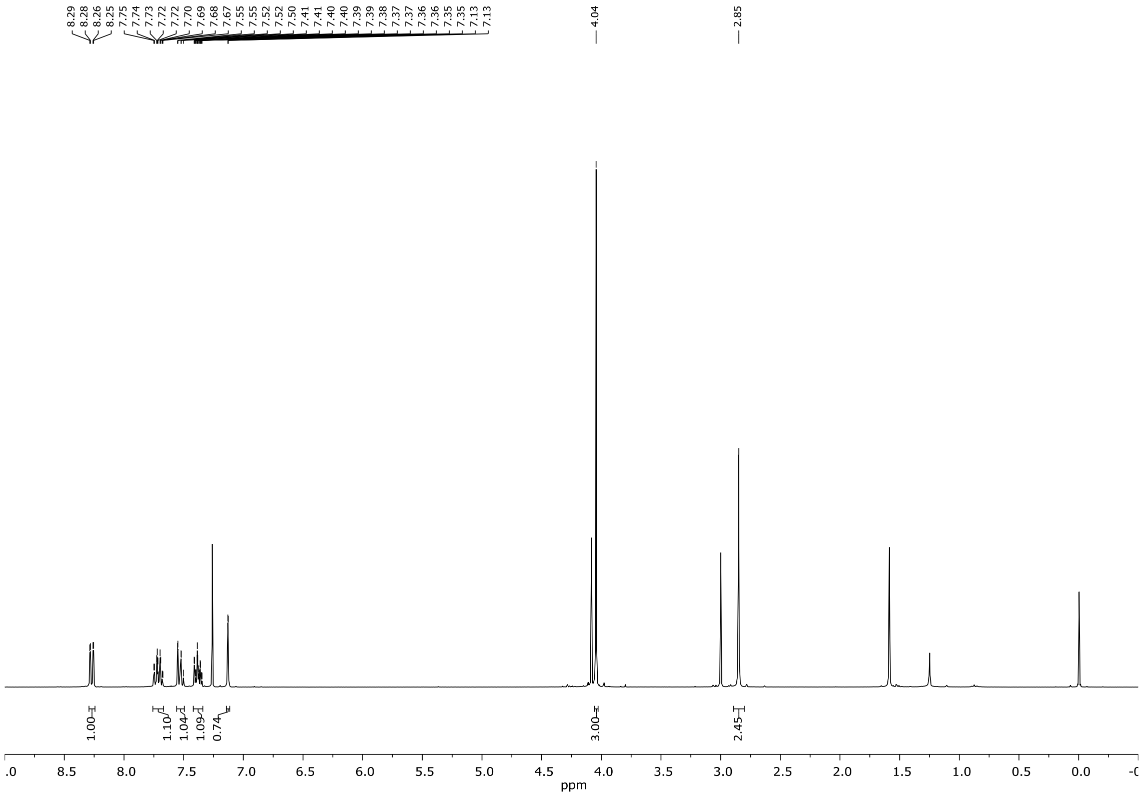

Figure S9. ^{1}H NMR spectrum of 3-hydroxy-4-methoxy-1-methyl-9*H*-xanthen-9-one (**10**) ($CDCl_3$, 300 MHz).

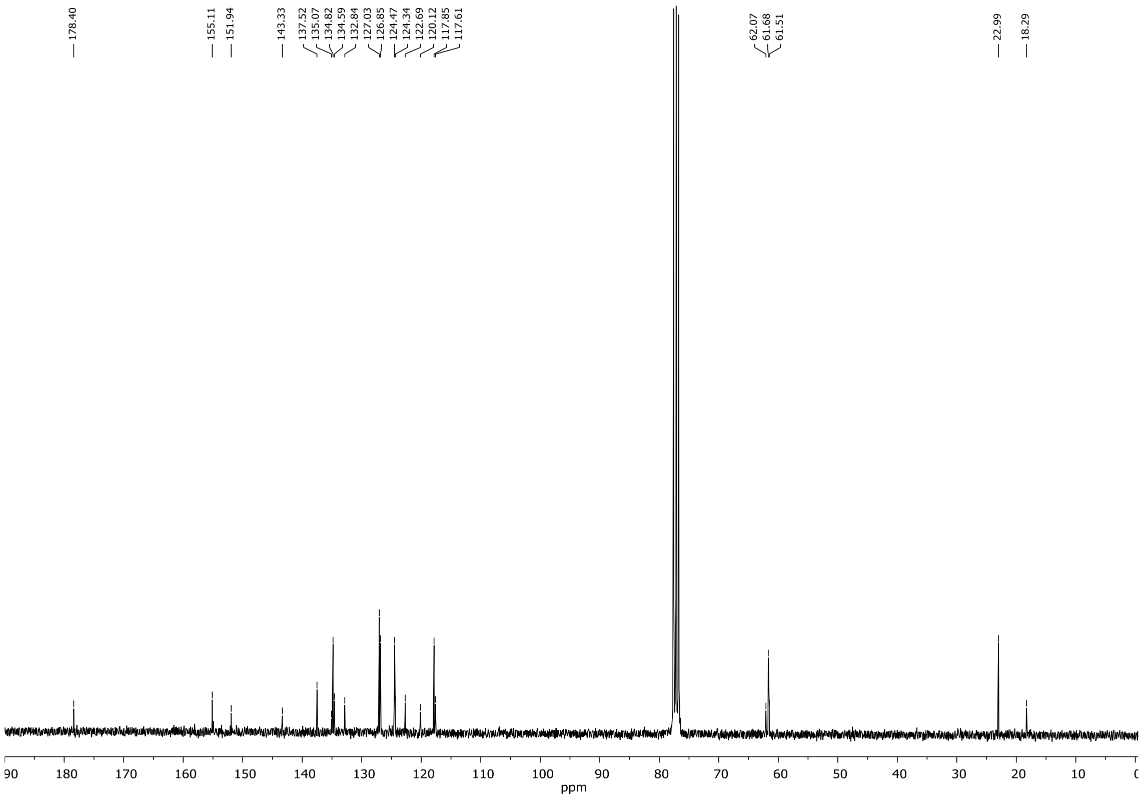

Figure S10. ^{13}C NMR spectrum of 3-hydroxy-4-methoxy-1-methyl-9*H*-xanthen-9-one (**10**) ($CDCl_3$, 300 MHz).

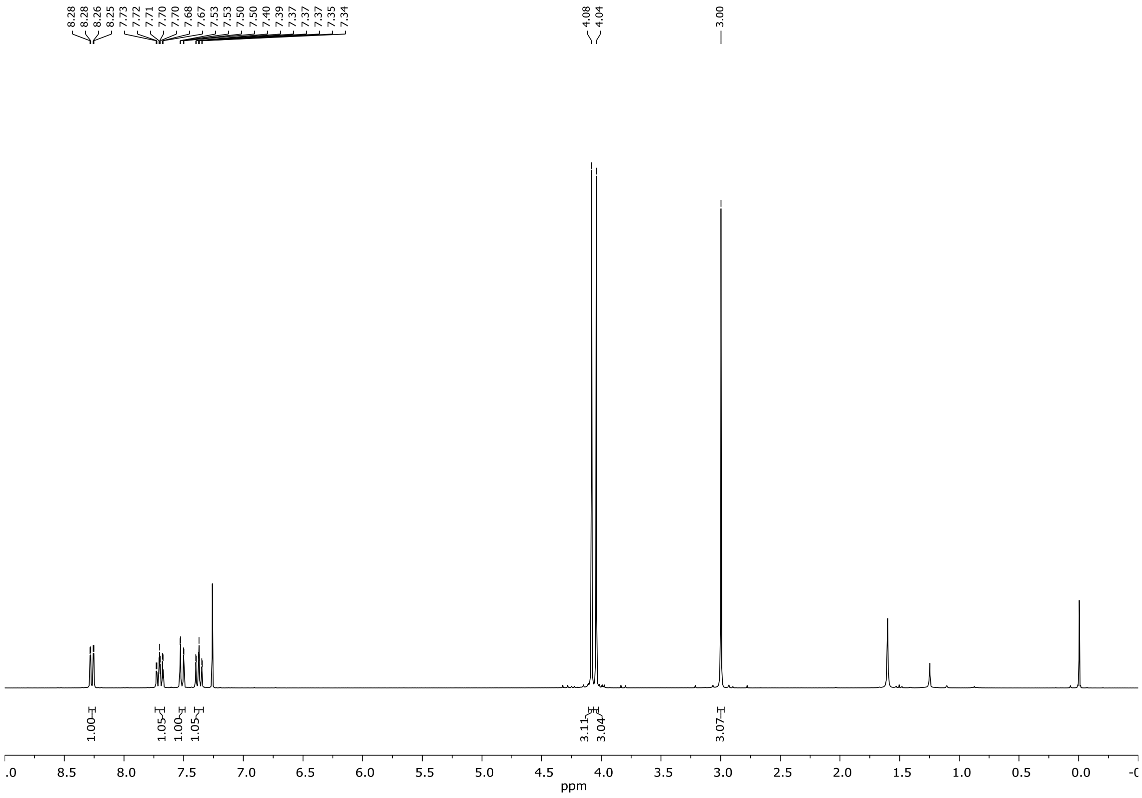

Figure S11. ^{1}H NMR spectrum of 2-chloro-3,4-dimethoxy-1-methyl-9*H*-xanthen-9-one (**11**) ($CDCl_3$, 300 MHz).

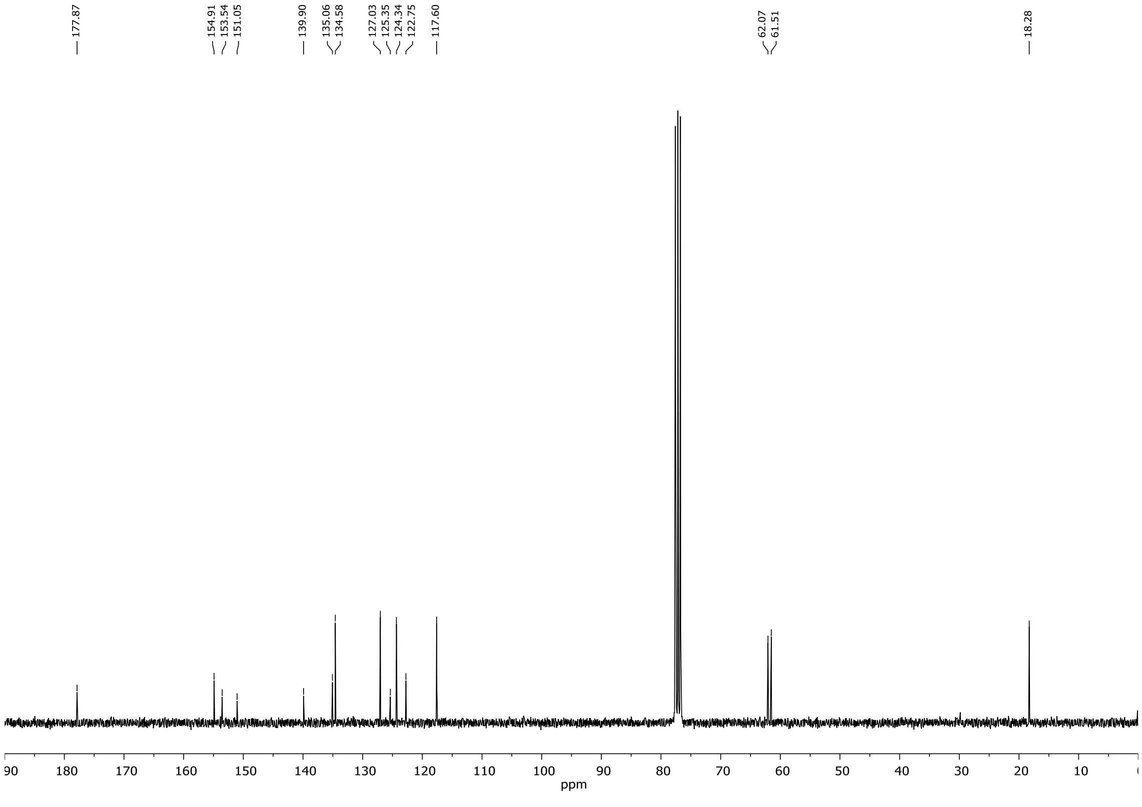

Figure S12. ^{13}C NMR spectrum of 2-chloro-3,4-dimethoxy-1-methyl-9*H*-xanthen-9-one (**11**) ($CDCl_3$, 300 MHz).

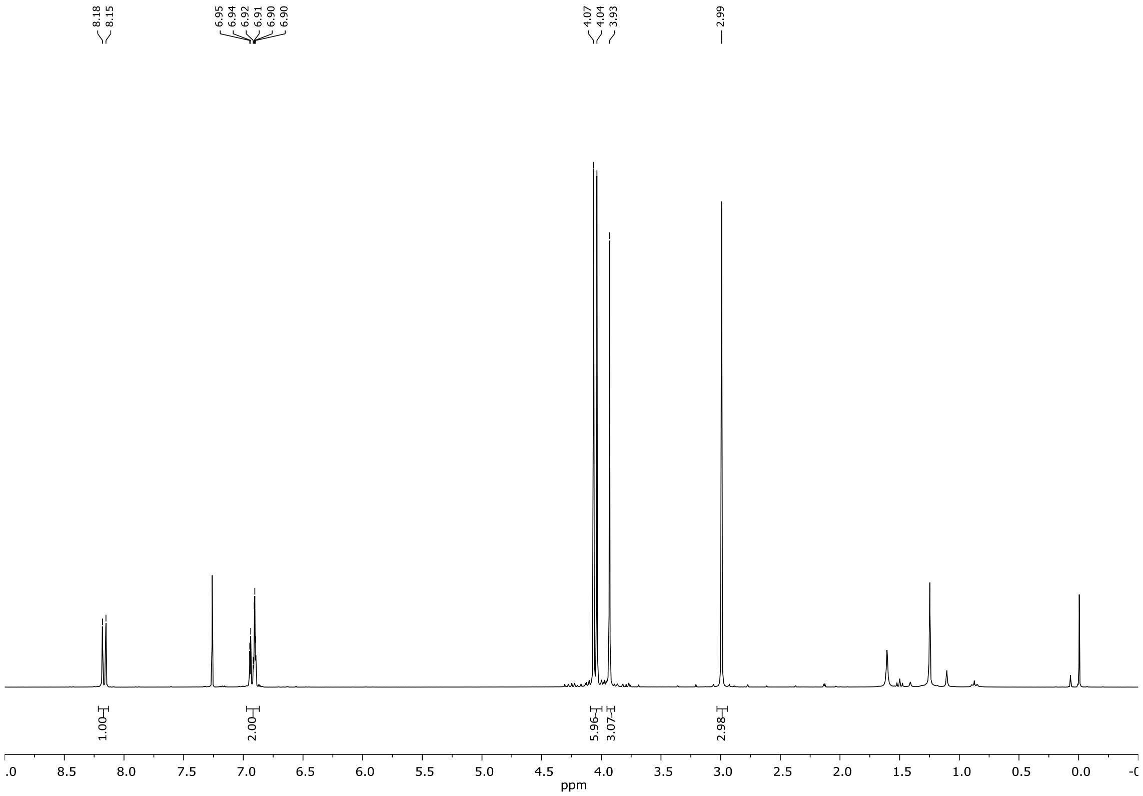

Figure S13. ^{1}H NMR spectrum of 2-chloro-3,4,6-trimethoxy-1-methyl-9*H*-xanthen-9-one (**12**) ($CDCl_3$, 300 MHz).

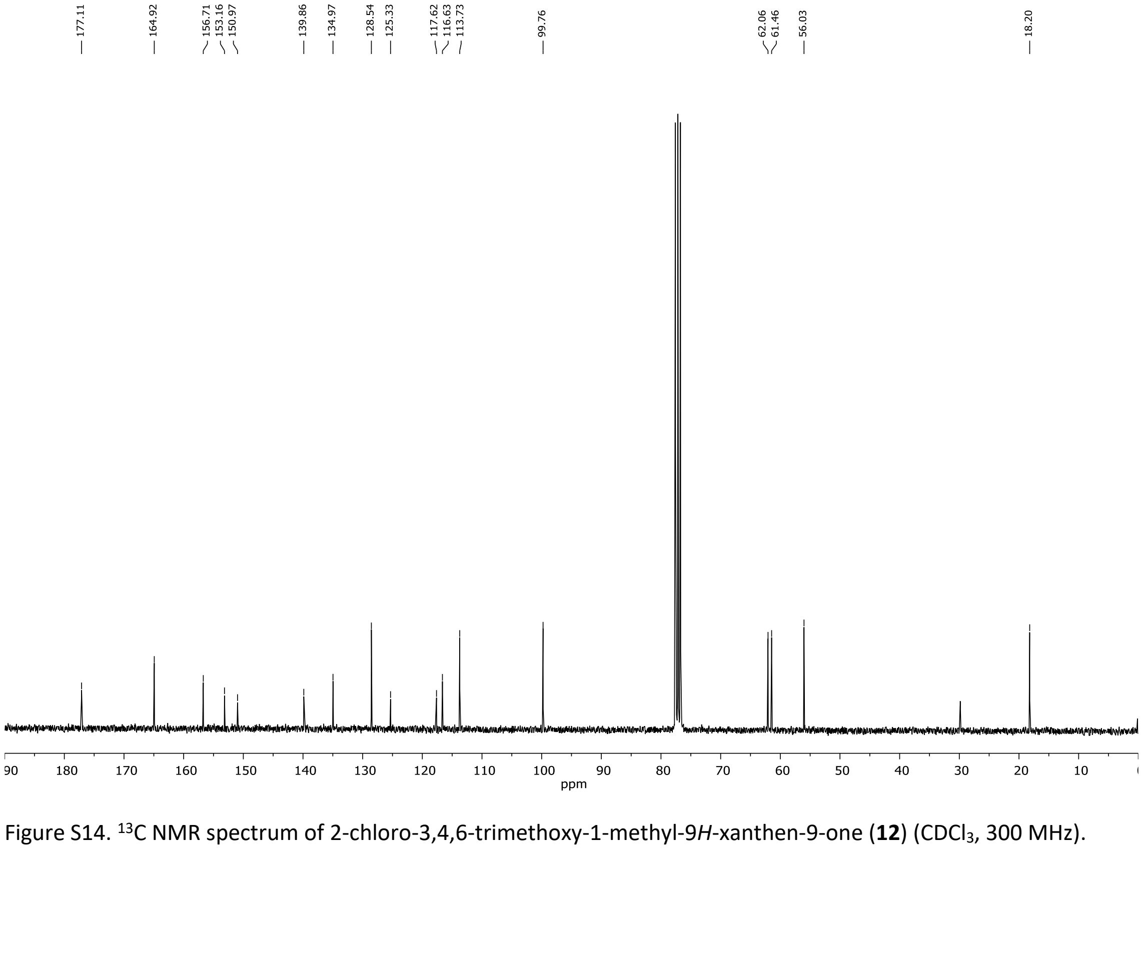

Figure S14. ^{13}C NMR spectrum of 2-chloro-3,4,6-trimethoxy-1-methyl-9*H*-xanthen-9-one (**12**) ($CDCl_3$, 300 MHz).

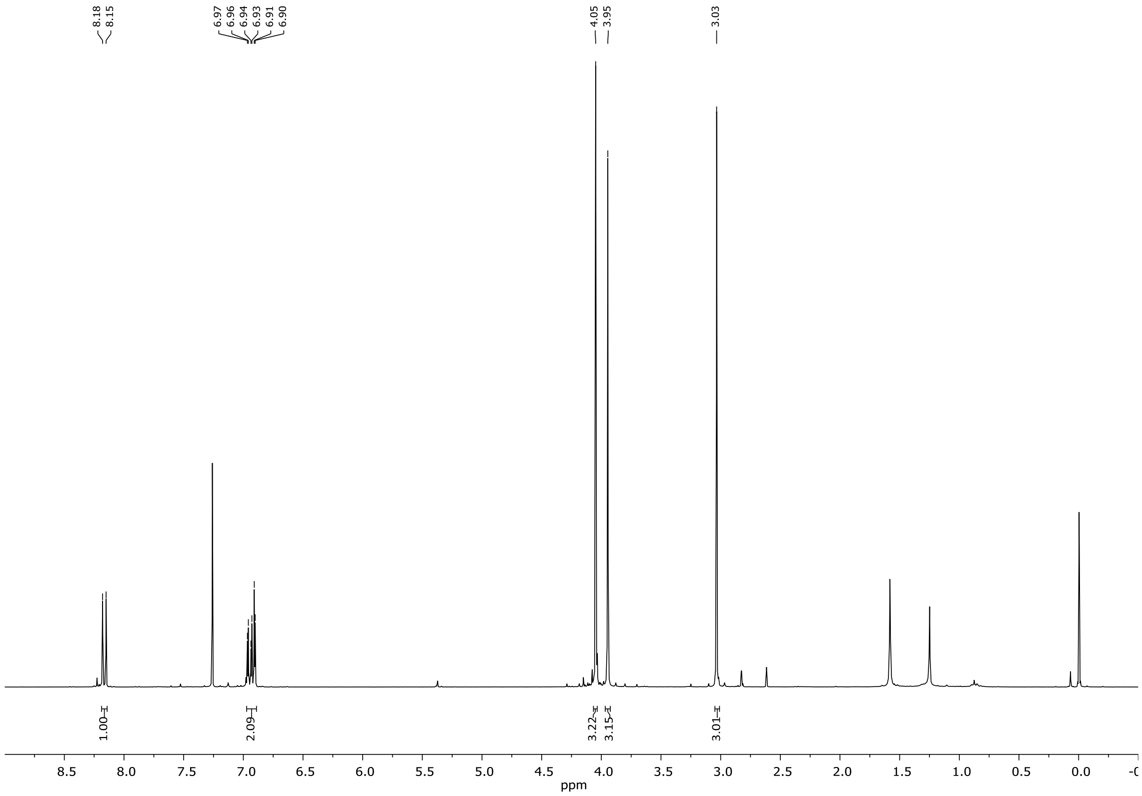

Figure S15. ^{1}H NMR spectrum of 2,3-dichloro-4,6-dimethoxy-1-methyl-9*H*-xanthen-9-one (**13**) ($CDCl_3$, 300 MHz).

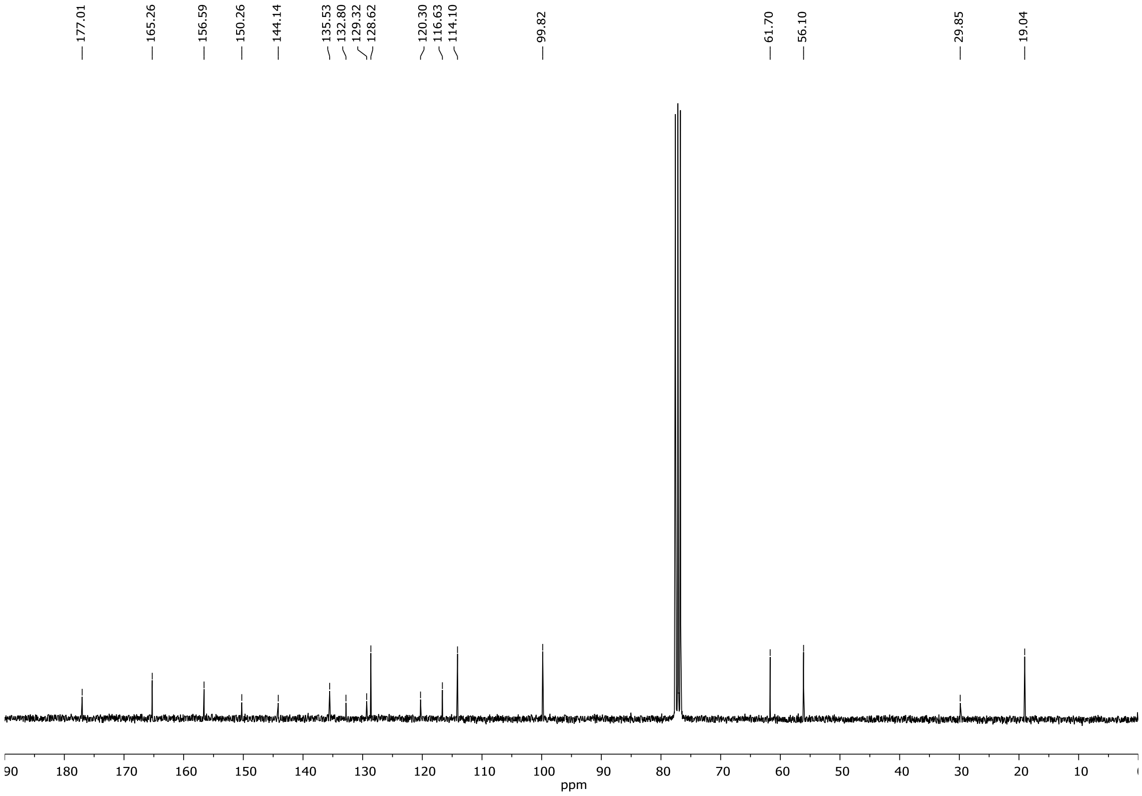

Figure S16. ^{13}C NMR spectrum of 2,3-dichloro-4,6-dimethoxy-1-methyl-9*H*-xanthen-9-one (**13**) ($CDCl_3$, 300 MHz).

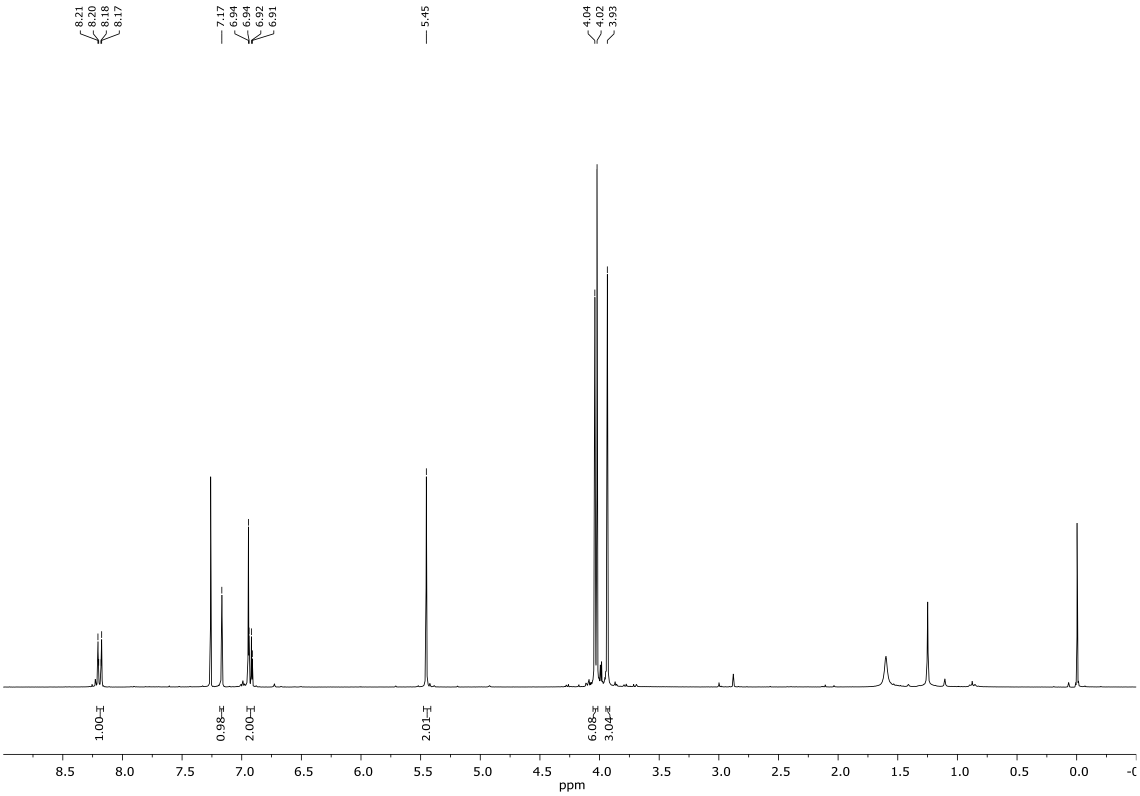

Figure S17. ^{1}H NMR spectrum of 1-(chloromethyl)-3,4,6-trimethoxy-9*H*-xanthen-9-one (**14**) ($CDCl_3$, 300 MHz).

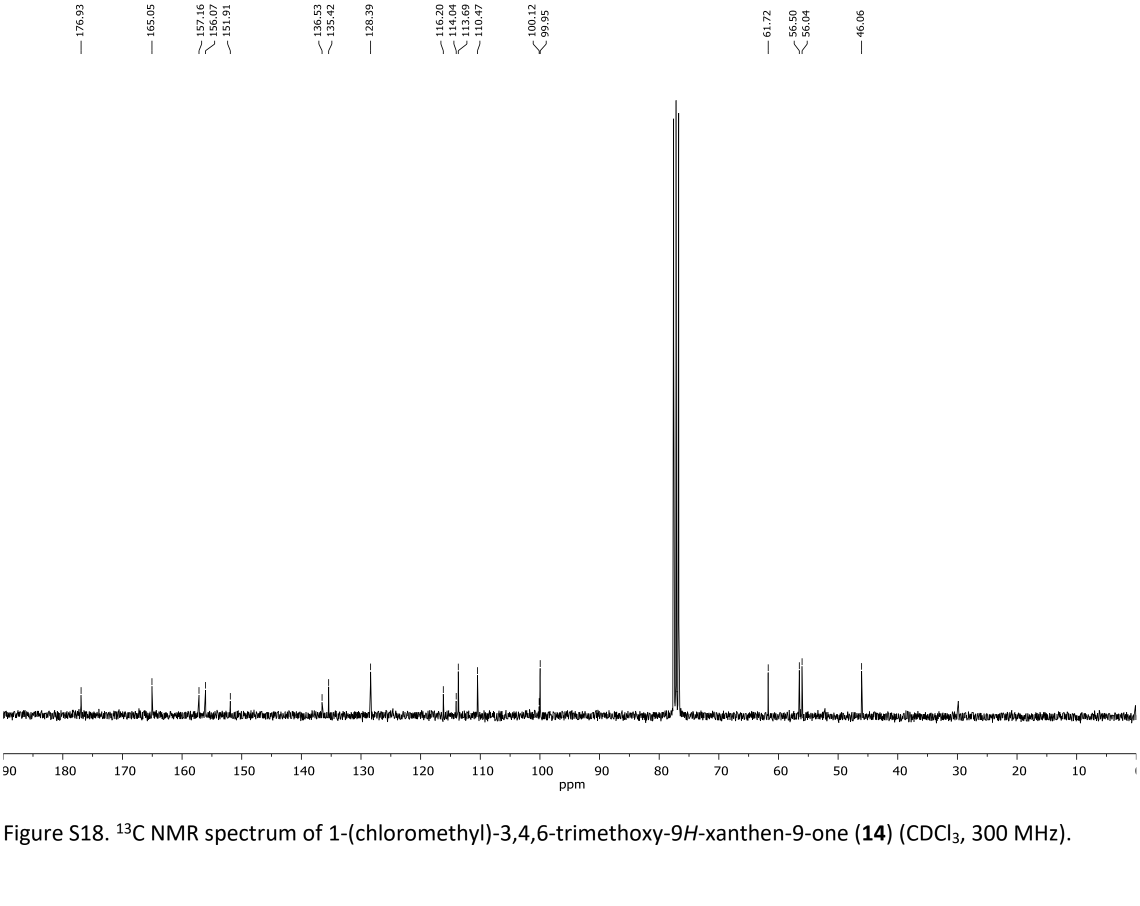

Figure S18. ^{13}C NMR spectrum of 1-(chloromethyl)-3,4,6-trimethoxy-9*H*-xanthen-9-one (**14**) ($CDCl_3$, 300 MHz).

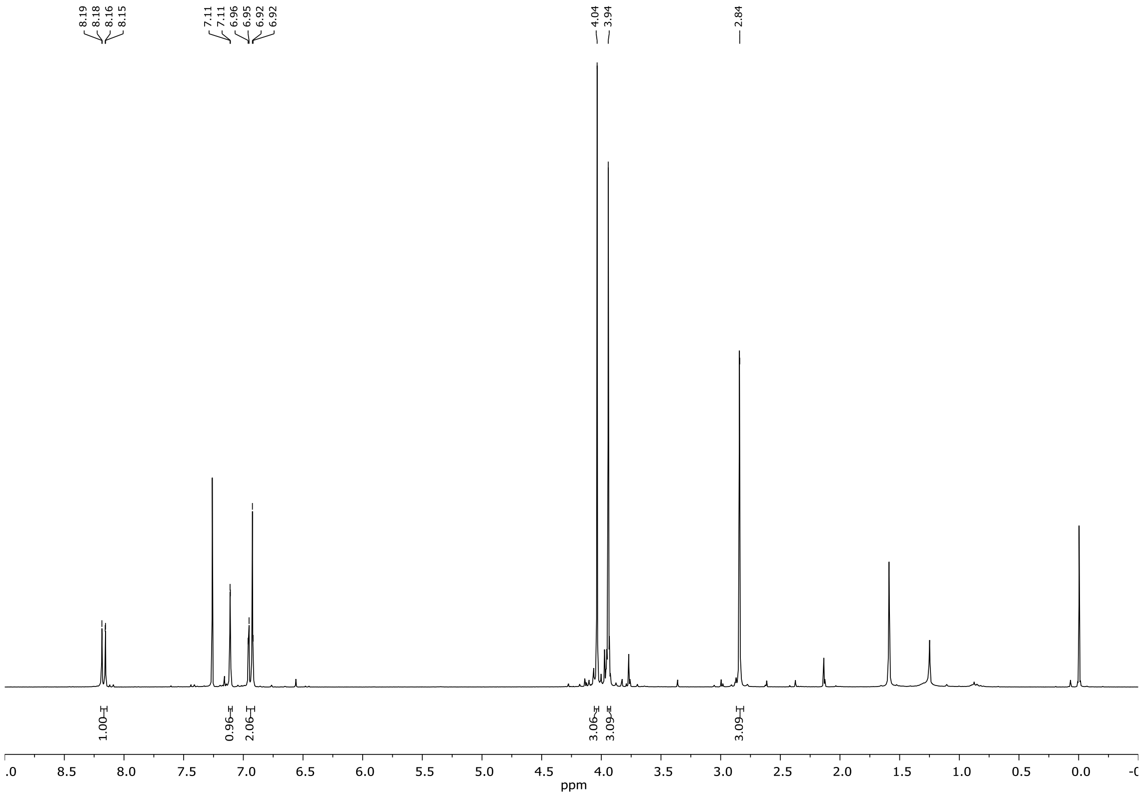

Figure S19. ^{1}H NMR spectrum of 3-chloro-4,6-dimethoxy-1-methyl-9*H*-xanthen-9-one (**15**) ($CDCl_3$, 300 MHz).

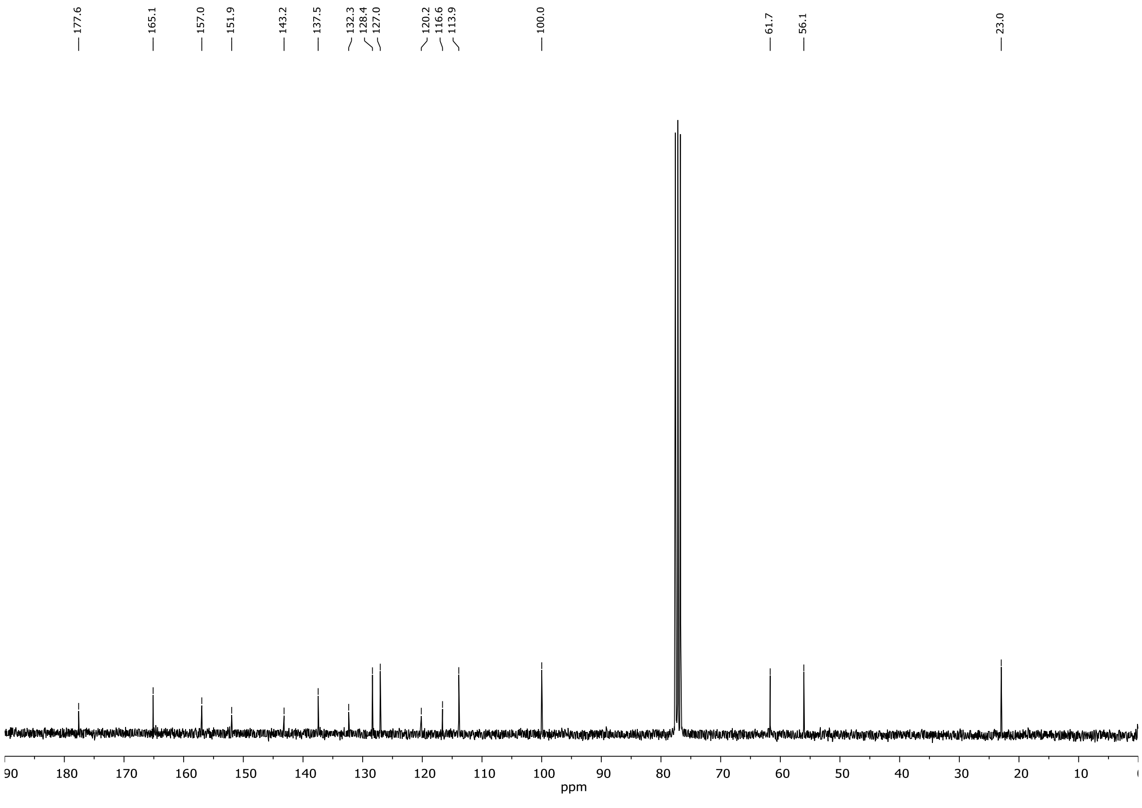

Figure S20. ^{13}C NMR spectrum of 3-chloro-4,6-dimethoxy-1-methyl-9*H*-xanthen-9-one (**15**) ($CDCl_3$, 300 MHz).

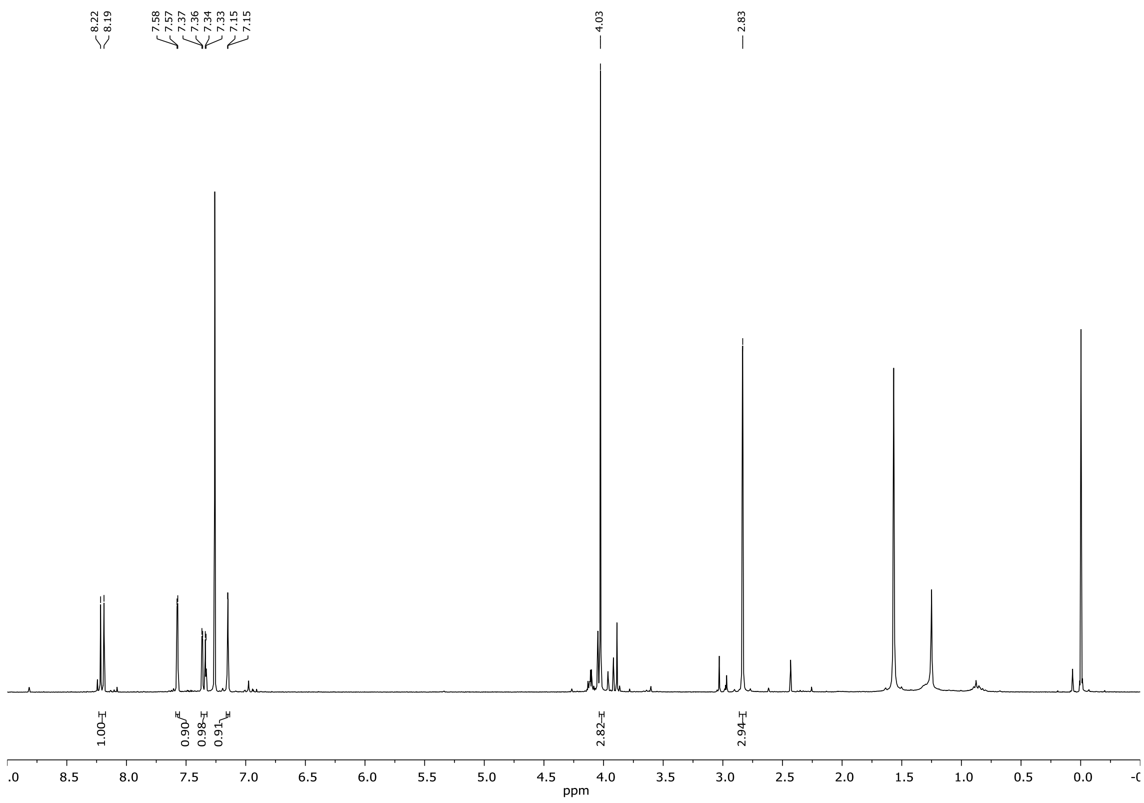

Figure S21. ^{1}H NMR spectrum of 3,6-dichloro-4-methoxy-1-methyl-9*H*-xanthen-9-one (**16**) ($CDCl_3$, 300 MHz).

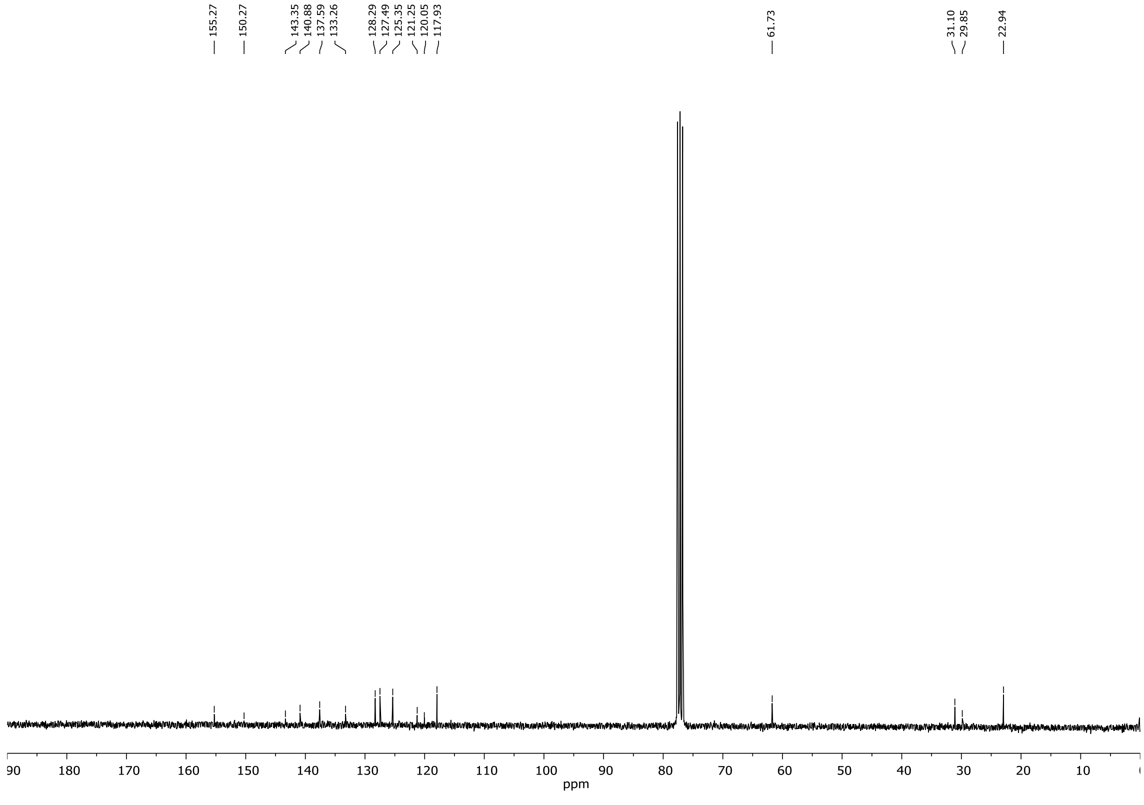

Figure S22. ^{13}C NMR spectrum of 3,6-dichloro-4-methoxy-1-methyl-9*H*-xanthen-9-one (**16**) ($CDCl_3$, 300 MHz).

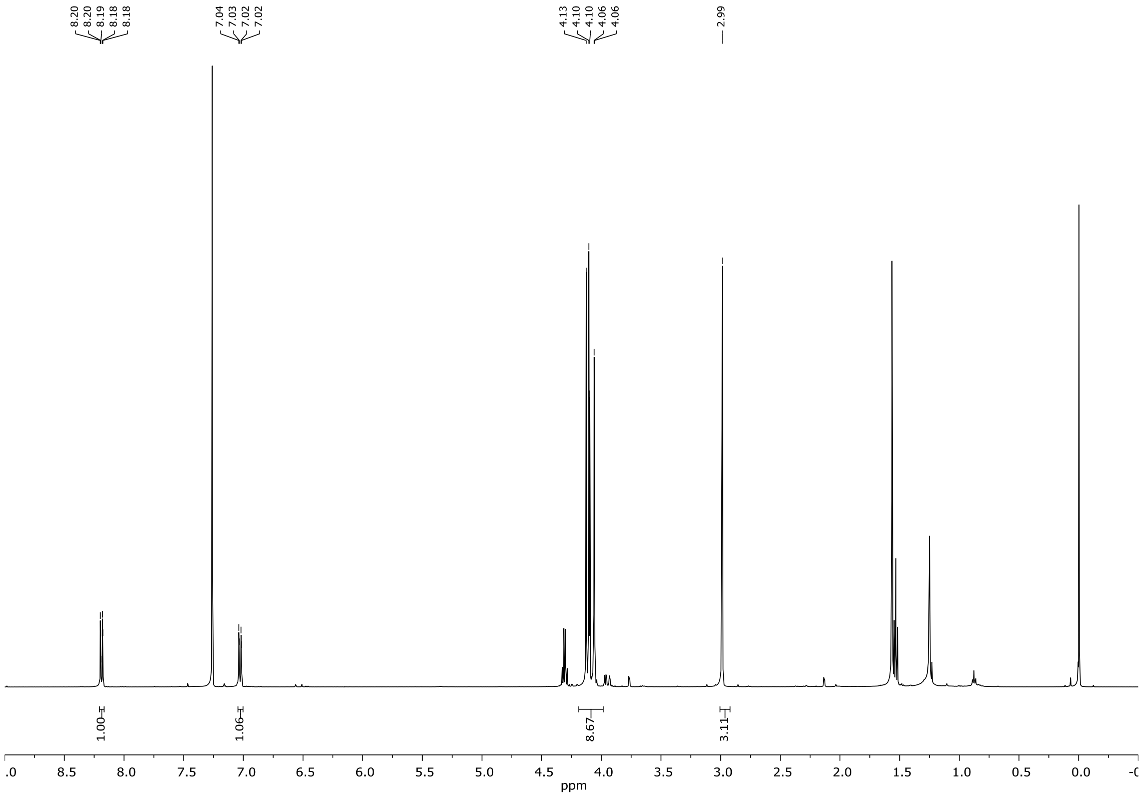

Figure S23. ^{1}H NMR spectrum of 2,5-dichloro-3,4,6-trimethoxy-1-methyl-9*H*-xanthen-9-one (**17**) ($CDCl_3$, 300 MHz).

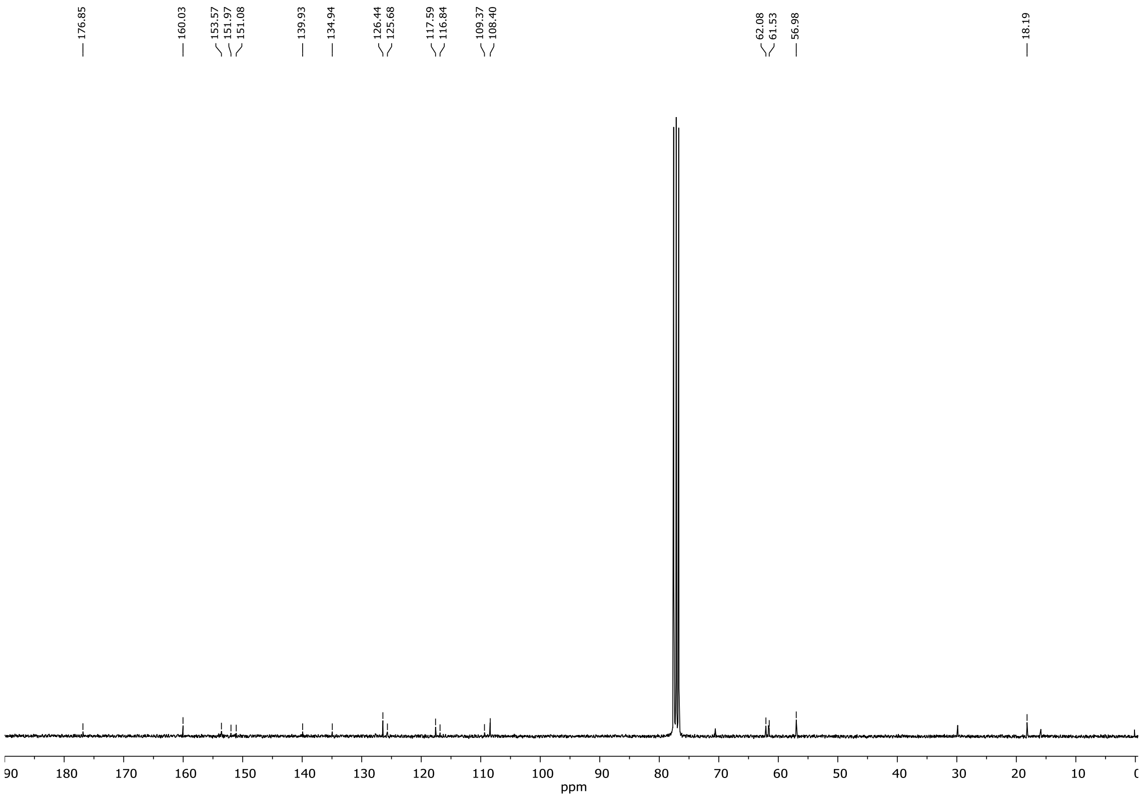

Figure S24. ^{13}C NMR spectrum of 2,5-dichloro-3,4,6-trimethoxy-1-methyl-9*H*-xanthen-9-one (**17**) ($CDCl_3$, 300 MHz).

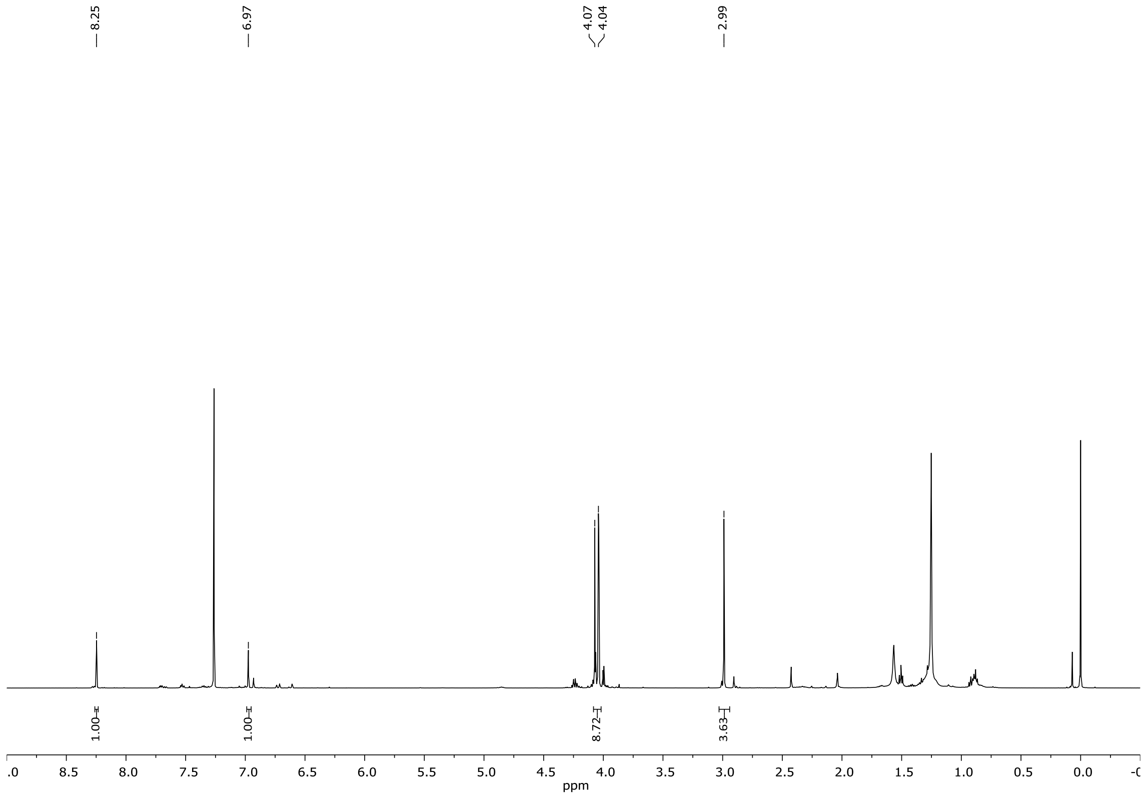

Figure S25. ^{1}H NMR spectrum of 2,7-dichloro-3,4,6-trimethoxy-1-methyl-9*H*-xanthen-9-one (**18**) ($CDCl_3$, 300 MHz).

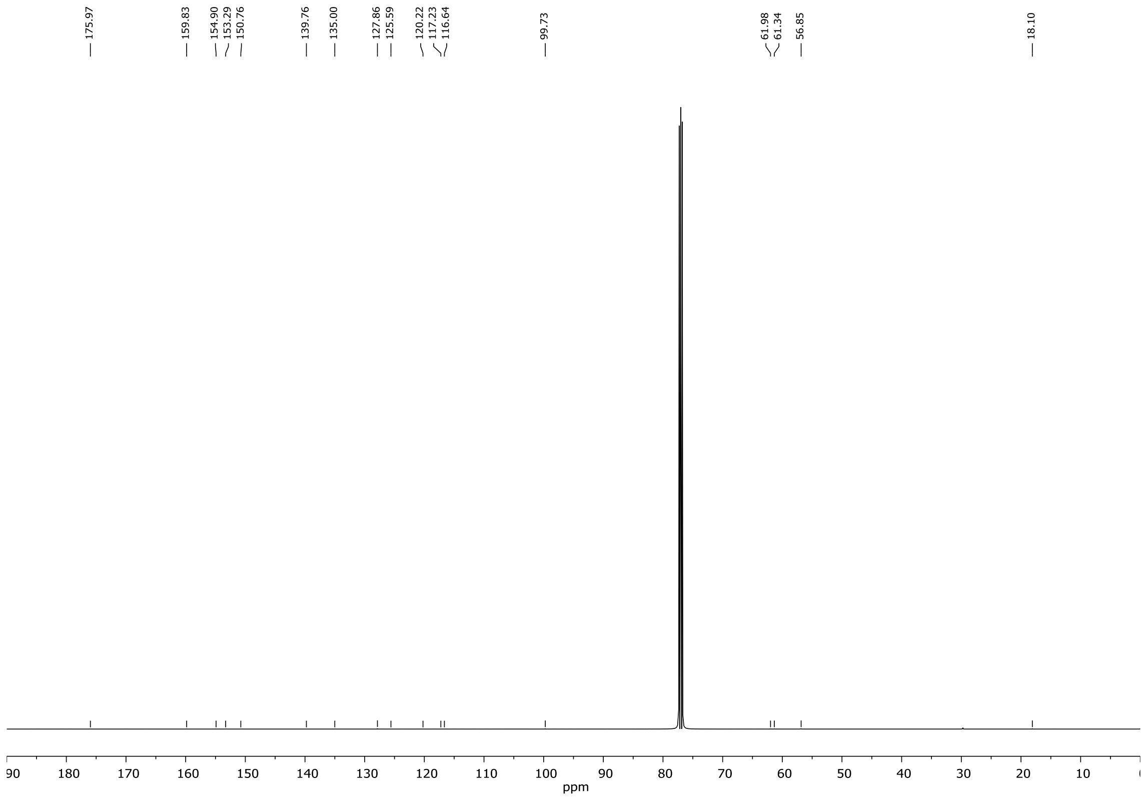

Figure S26. ^{13}C NMR spectrum of 2,7-dichloro-3,4,6-trimethoxy-1-methyl-9*H*-xanthen-9-one (**18**) ($CDCl_3$, 300 MHz).

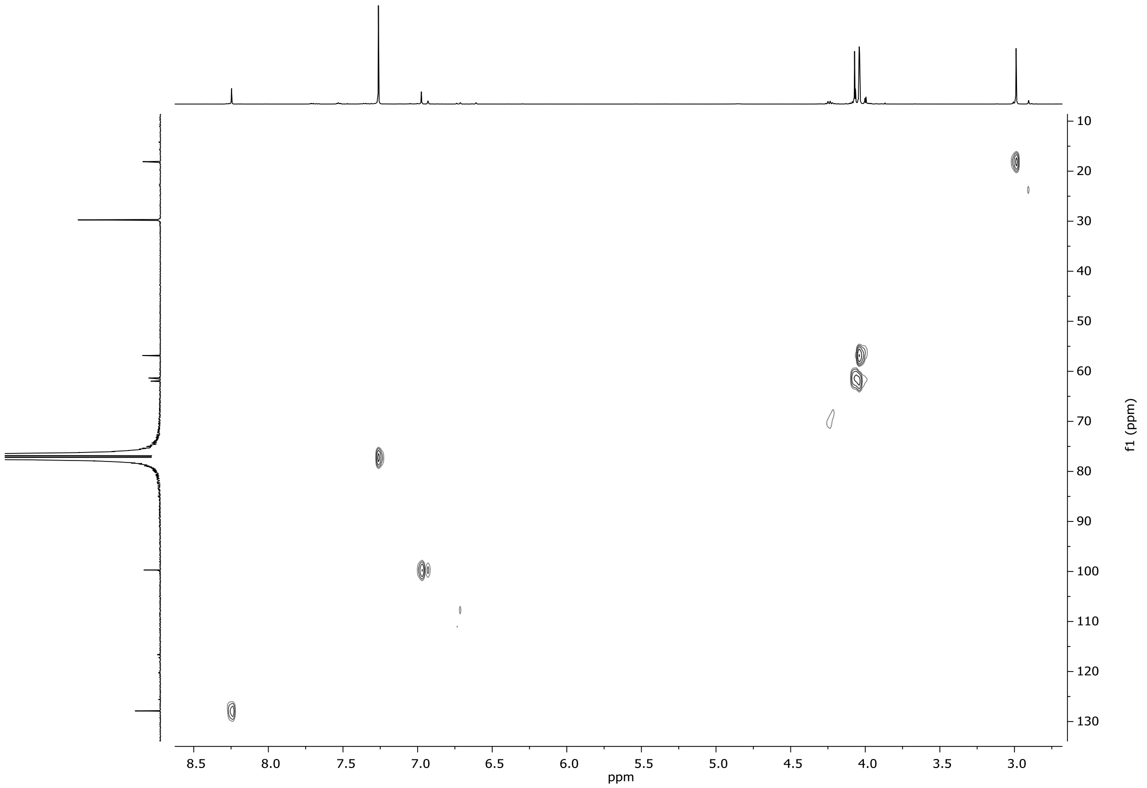

Figure S27. HSQC spectrum of 2,7-dichloro-3,4,6-trimethoxy-1-methyl-9*H*-xanthen-9-one (**18**) ($CDCl_3$, 300 MHz).

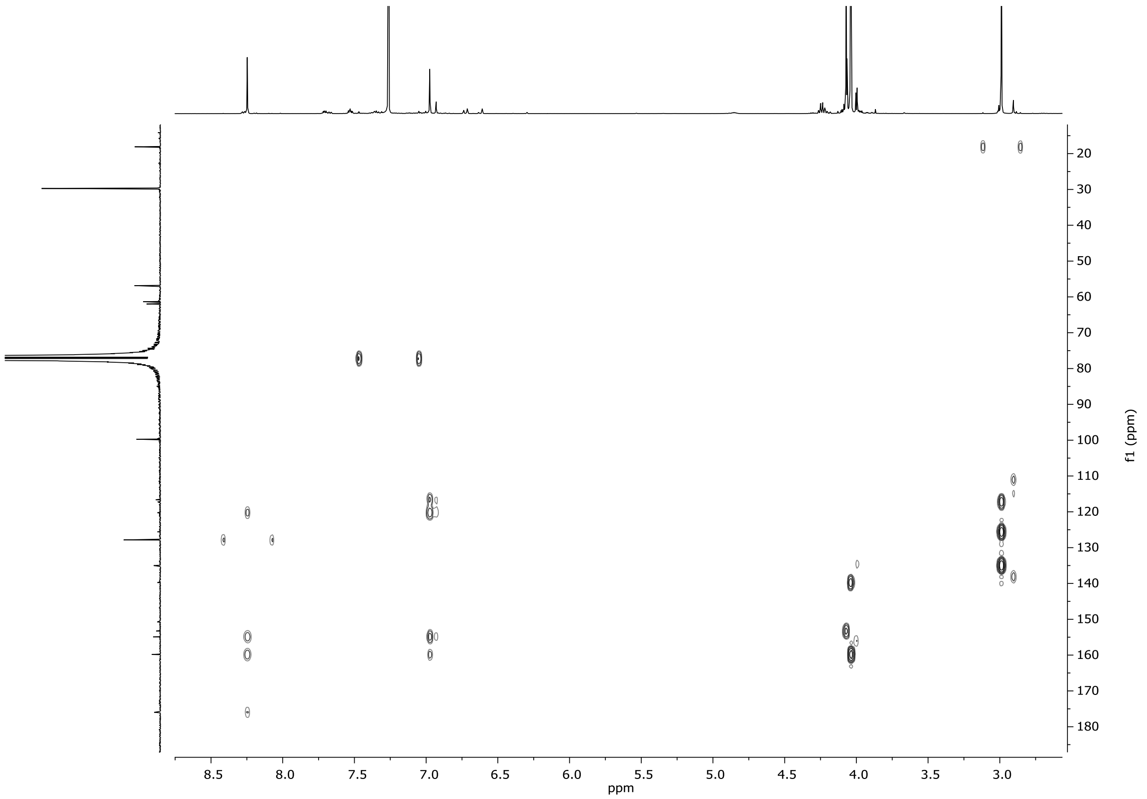

Figure S28. HMBC spectrum of 2,7-dichloro-3,4,6-trimethoxy-1-methyl-9*H*-xanthen-9-one (**18**) ($CDCl_3$, 300 MHz).

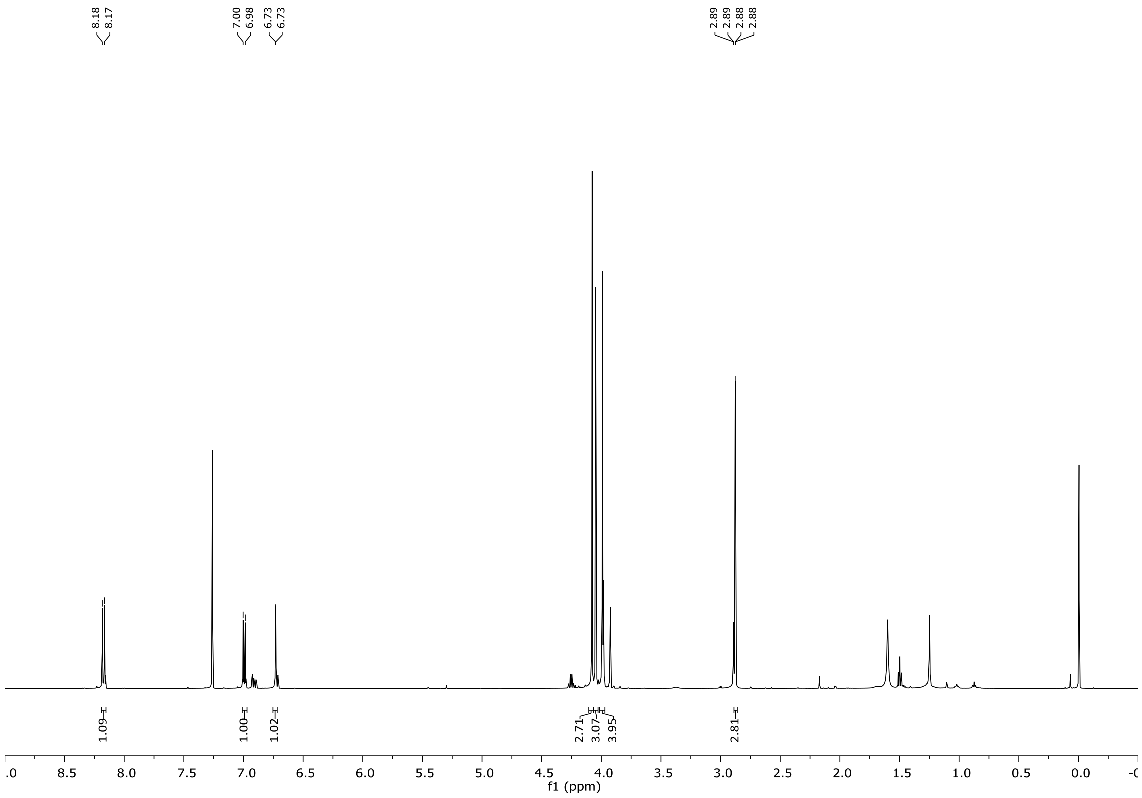

Figure S29. ^{1}H NMR spectrum of 5-chloro-3,4,6-trimethoxy-1-methyl-9*H*-xanthen-9-one (**19**) ($CDCl_3$, 300 MHz).

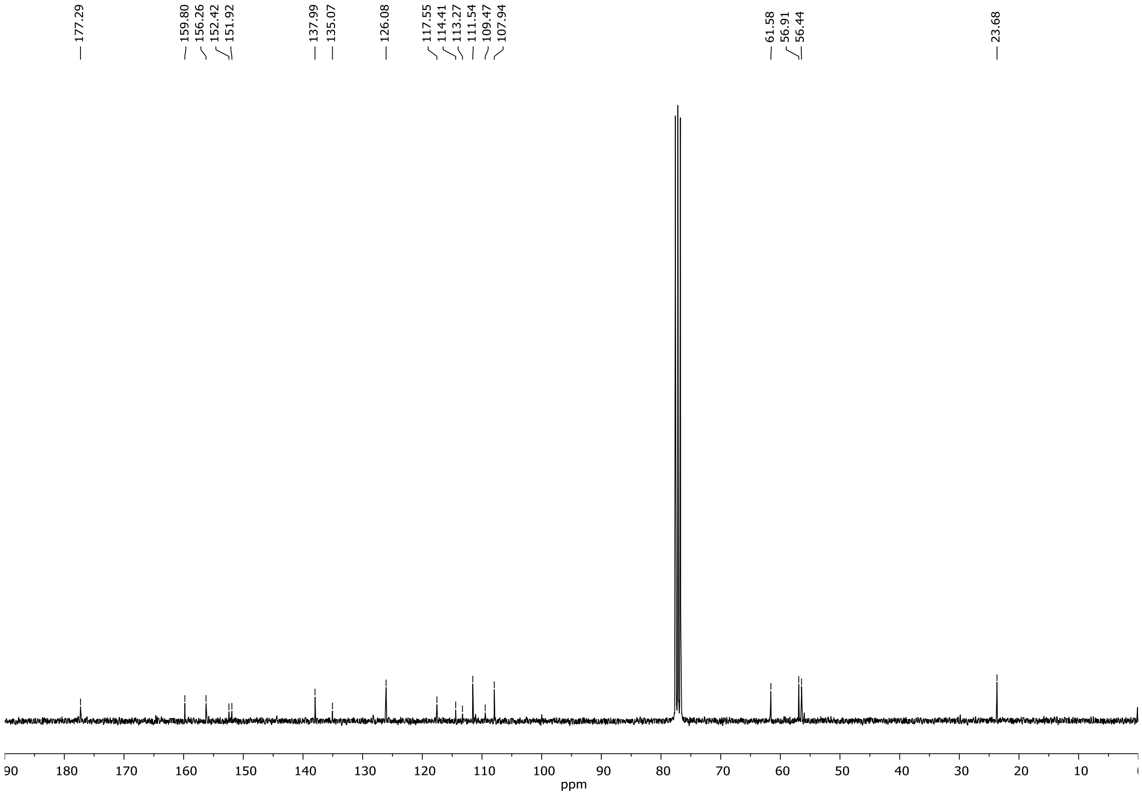

Figure S30. ^{13}C NMR spectrum of 5-chloro-3,4,6-trimethoxy-1-methyl-9*H*-xanthen-9-one (**19**) ($CDCl_3$, 300 MHz).

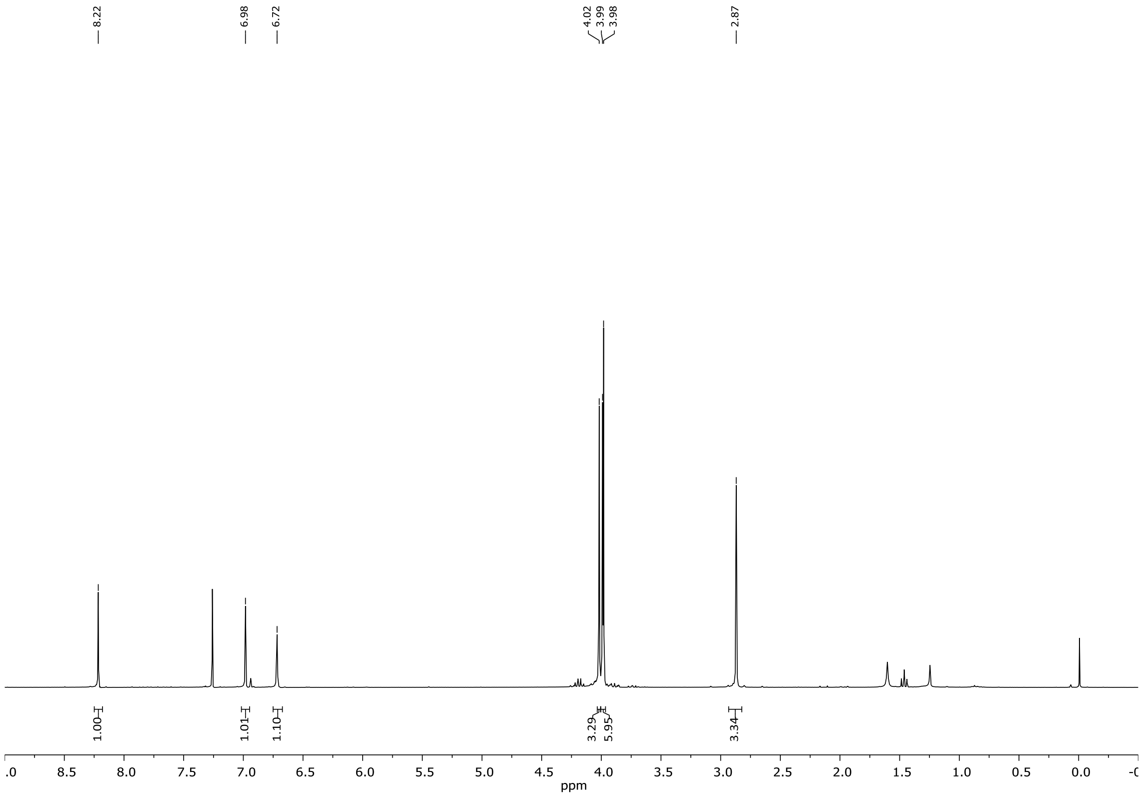

Figure S31. ^{1}H NMR spectrum of 7-chloro-3,4,6-trimethoxy-1-methyl-9*H*-xanthen-9-one (**20**) ($CDCl_3$, 300 MHz).

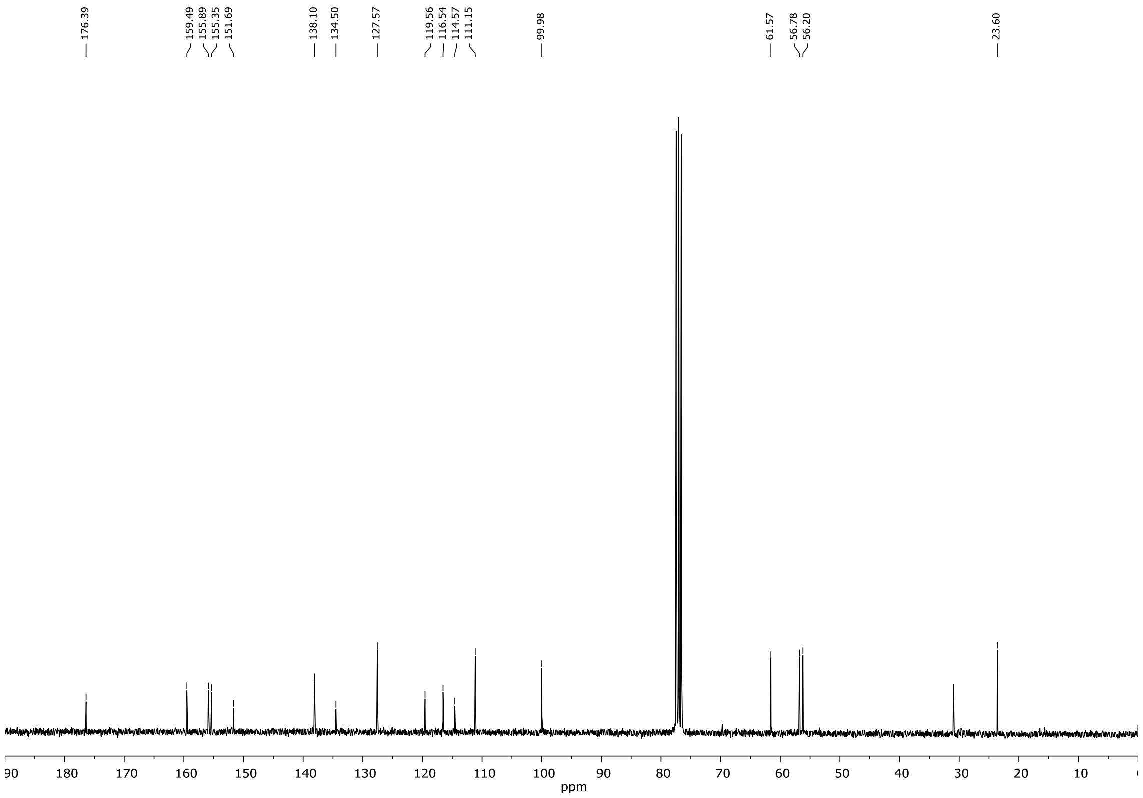

Figure S32. ^{13}C NMR spectrum of 7-chloro-3,4,6-trimethoxy-1-methyl-9*H*-xanthen-9-one (**20**) ($CDCl_3$, 300 MHz).
